# A Mismatch-Based Model for Memory Reconsolidation and Extinction in Attractor Networks

**DOI:** 10.1371/journal.pone.0023113

**Published:** 2011-08-03

**Authors:** Remus Osan, Adriano B. L. Tort, Olavo B. Amaral

**Affiliations:** 1 Center for Neuroscience, Boston University, Boston, Massachusetts, United States of America; 2 Center for Biodynamics, Boston University, Boston, Massachusetts, United States of America; 3 Department of Mathematics and Statistics, Boston University, Boston, Massachusetts, United States of America; 4 Brain Institute, Federal University of Rio Grande do Norte, Natal, Rio Grande do Norte, Brazil; 5 Edmond and Lily Safra International Institute of Neuroscience of Natal, Natal, Rio Grande do Norte, Brazil; 6 Institute of Medical Biochemistry, Federal University of Rio de Janeiro, Rio de Janeiro, Brazil; Georgia State University, United States of America

## Abstract

The processes of memory reconsolidation and extinction have received increasing attention in recent experimental research, as their potential clinical applications begin to be uncovered. A number of studies suggest that amnestic drugs injected after reexposure to a learning context can disrupt either of the two processes, depending on the behavioral protocol employed. Hypothesizing that reconsolidation represents updating of a memory trace in the hippocampus, while extinction represents formation of a new trace, we have built a neural network model in which either simple retrieval, reconsolidation or extinction of a stored attractor can occur upon contextual reexposure, depending on the similarity between the representations of the original learning and reexposure sessions. This is achieved by assuming that independent mechanisms mediate Hebbian-like synaptic strengthening and mismatch-driven labilization of synaptic changes, with protein synthesis inhibition preferentially affecting the former. Our framework provides a unified mechanistic explanation for experimental data showing (a) the effect of reexposure duration on the occurrence of reconsolidation or extinction and (b) the requirement of memory updating during reexposure to drive reconsolidation.

## Introduction

The concept of memory reconsolidation was proposed more than 40 years ago [1], but has recently regained considerable attention in the literature [2]. Most of the data in favor of the reconsolidation hypothesis has stemmed from the finding that pharmacological agents can induce amnesia when administered after reexposure to a context in which a memory was originally learned [3], [4]. This finding initially sparked controversy, as studies of memory extinction had traditionally found a directly opposite effect: namely, that the same drugs could block extinction, therefore preserving the original memory [5], [6].

A number of studies later tried to reconcile these apparently paradoxical effects, showing that both phenomena are possible outcomes of nonreinforced reexposure, and that the occurrence of one or another depends on the experimental protocol: in conditions in which extinction is observed in controls, amnestic drugs block extinction and preserve the original memory; meanwhile, in conditions causing no extinction, the same drugs cause amnesia, putatively due to disruption of reconsolidation [7], [8]. These results led to the proposition that the “dominant trace” after reexposure is the one made labile to amnestic agents [7].

The fact that not all studies could demonstrate reconsolidation by post-reexposure interventions [9], [10] also suggested that there are “boundary conditions” which are necessary for trace labilization [11], [12]. One of these conditions has been proposed to be the occurrence of memory updating during reexposure [13], due to studies in which simple reexposure in the absence of new information did not lead to reconsolidation, as shown by the lack of effect of amnestic drugs [14], [15]. Similarly, other studies have shown that very short reexposure trials were also associated with no effect of these drugs [8], [16].

Understanding what determines the occurrence of these phenomena is important, as modulations of both reconsolidation and extinction have begun to be tested as therapeutic strategies in anxiety disorders such as PTSD [17] and phobias [18]. To date, no mechanism has been postulated to explain how changes in a single variable such as reexposure duration can lead to these different outcomes. Since the same drugs can block (or enhance) both reconsolidation and extinction, however, it is feasible to hypothesize that the differences between these processes depend not only on their molecular features, but also – and perhaps mainly – on their network properties.

Attractor network models have provided a general framework through which information storage can be modeled in connected networks, and the existence of attractors in brain structures such as the hippocampus [19], [20], neocortex [21] and olfactory bulb [22] has received experimental support from electrophysiological studies. By assuming that memory processing is based on attractor dynamics, and that updating of a memory trace occurs based on mismatch-induced synaptic changes, we propose a model which can explain how contextual reexposure may lead to reconsolidation or extinction. In this framework, the dominant process occurring after reexposure depends on the degree of mismatch between the animal's current representation of a context and a previously stored attractor. The model accounts for the different effects of amnestic agents on reconsolidation and extinction, as well as for the requirement of dissimilarities between the learning and reexposure sessions for reconsolidation to occur.

## Results

### Model Framework

To study the processes described above computationally, we use an adaptation of the classical attractor network model [23], [24]. These highly connected neural networks, which can store memories as neuronal activation patterns based on Hebbian modifications of synaptic weights, have been proposed to be simple correlates of autoassociative networks such as the one believed to exist in region CA3 of the hippocampus [25], [26]. Attractor-like functioning has been shown to be compatible with both firing-rate and spike-time dependent plasticity in spiking neuronal networks [27], [28]. For the sake of simplicity, however, and for better correlation with previous models dealing with the effect of mismatch and memory representations (e.g. [29]), we use the classical firing rate implementation, which remains a useful tool for studying emergent network properties related to learning and memory.

**Figure 1:**
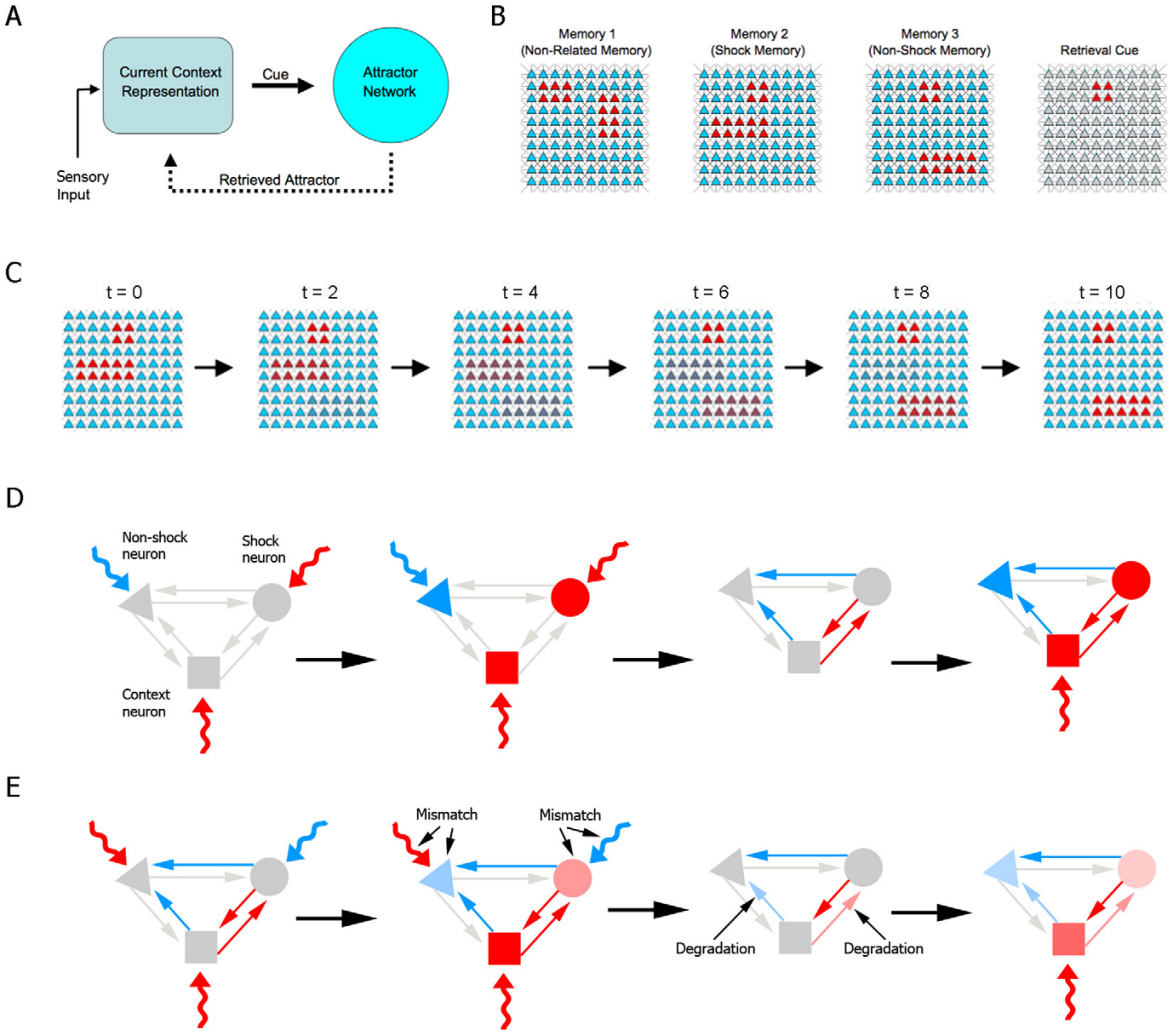
Model description. (**A**) General scheme of the model architecture. (**B**) Patterns of 100 neurons used to represent memory 1 (unrelated memory), memory 2 (shock memory), memory 3 (non-shock memory) and retrieval cue. Red denotes activation, while blue denotes inhibition. Shock and non-shock memories share the activation of context neurons (red square), which are used to test retrieval. (**C**) Transformation of cue pattern according to reexposure duration. For short durations (low *t* values), the pattern resembles the shock memory (left), while cues for long durations (high *t* values) resemble the non-shock memory (right) and intermediate durations (center) yield mixed patterns. (**D**) Retrieval-induced synthesis in fear learning. Activation of shock (circle) and context (square) neurons is driven by excitatory cues (red curved arrows), while inhibition of a non-shock neuron (triangle) is caused by an inhibitory cue (blue curved arrow). This leads to the establishment of excitatory/inhibitory synaptic weights between neurons (red/blue arrows), which allow reinstatement of the pattern by presentation of the context (right panel). (**E**) Mismatch-induced degradation in nonreinforced reexposure. A cue exciting non-shock and context neurons and inhibiting shock neurons is presented (left panel), but retrieval of the shock pattern occurs due to previously established synaptic weights, causing mismatch between activation patterns in the cue and attractor networks. This leads to degradation of synaptic weights responsible for the mismatch, causing reinstatement of the shock pattern in response to context to be weakened in a subsequent test session (right panel).

Neuronal activities in the attractor network (meant to represent a hippocampal auto-associative storage network in our model) are determined by equation (1):
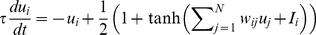
(1)where *τ* is the neural time constant and 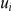 represents the level of activation of neuron *i* in a network comprised by *N* neuronal units, varying continuously from 0 to 1 for each neuron, and not from −1 to 1 as in classical formulations (see Methods). This can reflect the firing rate and connectivity of neurons in a more realistic way, as it solves a series of biologically unfeasible features of the original formulation, including (a) the requirement of symmetric connections between neurons, (b) the strengthening of connections between neurons with low activity and (c) the occasional retrieval of mirror patterns diametrically opposite to those originally learned. The term 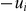 causes the activation level to decay towards 0, while the term 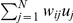 represents the influence of presynaptic neurons within the attractor network, weighed by the strength of the synaptic connections 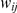. Finally, the term 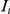 represents synaptic influences from cue inputs. These cue inputs are thought to represent cortical afferents providing the hippocampus with the animal's current representation of its environment, based both on external (i.e. sensory input) and internal information (i.e. retrieved memories) (Figure 1A). The interplay between sensory information and hippocampal feedback is not modeled explicitly; instead, the presented cues will be modeled as relying more on external or internal input depending on behavioral parameters (see below).

Learning in the model occurs through presentation of an activation pattern by the cue inputs, which leads to changes in the synaptic weight matrix 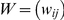, as determined by equation (2):
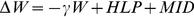
(2)where 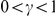 is a time-dependent synaptic decay factor [30], [31], and 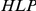 and 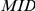 stand for *Hebbian Learning Plasticity* and *Mismatch-Induced Degradation*, respectively, expressed in array form. Both of these matrices are dependent on the steady state pattern of neuronal activation that is reached by the network upon cue presentation (Eq. (1)). The precise meaning of the 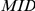 term and its equation will be explained below; for now, we will mention that all entries in the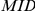 matrix are related to mismatch between the cue and a retrieved attractor and, as such, equal zero during initial learning. The 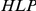 term represents a modified Hebbian learning factor (see Methods), and it is given by
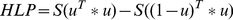
(3)where the vector 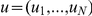 is the steady state of the network and 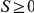 corresponds to a factor representing a sum of the biochemical requirements for Hebbian synaptic plasticity, such as receptor activation, intracellular signaling and protein synthesis. Thus, if two neurons are maximally active (*u_i_* = *u_j_* = 1), the *u_i_→u_j_* connection gets reinforced by *S*; if the presynaptic neuron *u_i_* is active and the postsynaptic neuron *u_j_* is silent (*u_j_* = 0), then the connection *u_i_→u_j_* changes by −*S*. If *u_i_* is silent, nothing happens to the connection *u_i_→u_j_*. Intermediate values of *u_i_* and *u_j_* lead to intermediate effects of these factors. The value of 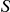 is what is modified in simulations studying the influence exerted by pharmacological agents on initial memory consolidation, reconsolidation and extinction. The effect of protein synthesis inhibition by anisomycin, for instance, is modeled by setting *S* to 0, thereby blocking Hebbian plasticity.

Training, reexposure and testing in a simple one-trial learning task, such as contextual fear conditioning, are modeled by setting up appropriate cue patterns. Training sessions consist of presentation of one of three complete patterns (Figure 1B): pattern 1, representing a memory which is unrelated to fear conditioning; pattern 2, representing fear conditioning training, in which a set of neurons representing the context is activated along with another set of neurons representing the presence of danger or an aversive stimulus (i.e. an electric shock); and pattern 3, representing fear conditioning extinction, in which the same context neurons are activated along with a different set of neurons representing absence of danger. The use of a specific pattern to represent extinction is motivated by experimental data suggesting that the extinction process represents the active learning of a new memory trace [5], [6], as well as by studies suggesting that it may be encoded by neuronal populations which are at least partially distinct from those involved in the original learning [32], [33].

Memory retrieval is tested by presenting the cue pattern that represents the context (Figure 1B), and observing the attractor to which the network evolves. We model the animal's behavioral response by assuming that retrieval of pattern 2 leads to a far greater degree of conditioned behavior in response to danger than when the network reaches another attractor (see Methods and Figure S1). In analogy to the experimental literature, we refer to the fear conditioned response as “freezing”, and use the percentage of time spent freezing during the test as a measure of memory in the task.

Nonreinforced reexposure to the context is modeled similarly to training, except that the cue pattern in this case is a mix of patterns 2 and 3. This is based on the assumption that, upon reexposure to the context in which fear learning occurred, the memory network will initially retrieve the aversive memory, with feedback from the hippocampus signaling the activation of neurons representing danger in the animal's contextual representation. Later within the trial, however, the absence of shock will lead the animal to start perceiving the context as non-threatening, with sensory information prevailing over the stored attractor and causing activation of neurons encoding the absence of danger. Thus, we assume that the animal's contextual representation changes gradually from pattern 2 to pattern 3 as the reexposure session becomes longer, leading the learning which occurs based on this session to become progressively more biased toward the new context rather than toward internal cues, as has been suggested to occur experimentally [34]. Therefore, the final cue pattern 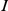 can vary from a pattern close to pattern 2 (for short reexposure times, in which the animal perceives the context as aversive throughout the session) to one close to pattern 3 (for long reexposure times, in which the animal perceives the context as non-threatening for most of the session), with reexposure times in between these two extremes yielding intermediate activation patterns (Figure 1C). In other words, we assume that the degree of mismatch between the final context representation during reexposure and the original representation formed upon initial learning is proportional to reexposure duration. We refer to this duration as *t*, and use a transformation from pattern 2 to pattern 3 which is a function of *t* to create the cue patterns representing different durations of reexposure (see Methods).

As in the initial training session, synaptic weights are updated after the reexposure session following equation (2), and the Hebbian learning rule acts by means of the *HLP* term (Figure 1D). However, the existence of a previously stored attractor for the context in the reexposure session can lead the memory network to retrieve an attractor which is different from the cue pattern employed, leading to mismatch between the two patterns. We thus introduce a memory updating system which degrades synaptic weights between the different sets of neurons responsible for this mismatch, reducing the strength of connections which cause disagreement with the new cue pattern (Figure 1E). This effect is modeled by the term *MID* in (2), which follows the equation:
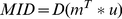
(4)where the *degradation factor D* represents biochemical requirements for mismatch-induced updating of synaptic connections – which are thought to involve, among other things, protein degradation [35], [36] – and 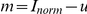 is the mismatch vector (where 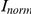 is a normalized cue vector varying between 0 and 1). Note that when the retrieved attractor is equal to the cue input (as during initial learning) there is no mismatch, since 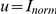 in these cases, leading all entries in vector *m* to equal zero.

Although the biochemical elements in the model are an obvious simplification (i.e. synaptic plasticity is certainly more complex than a synthesis/degradation balance, and involves many other mechanisms), there is much evidence to suggest that protein synthesis is a defining factor in long-term memory consolidation [37], as well as some evidence [35], [36] to suggest that protein degradation through the ubiquitin-proteasome system is involved in trace labilization during reconsolidation. Therefore, we focus on these two parameters in our simulations of pharmacological experiments. The synaptic weight changes induced by these processes are modeled as occurring during the post-reexposure period, based upon the activation state reached during the reexposure session (which presumably sets in motion the biochemical cascades and transcriptional information which will drive the protein changes occurring later). Pharmacological interventions after reexposure are thus modeled as changing either *S* or *D* during the synaptic weight updating process caused by the reexposure session (Eq. (2)), and the effects of these interventions are measured by evaluating subsequent retrieval in response to the cue representing the context.

### Learning and extinction in the model

**Figure 2:**
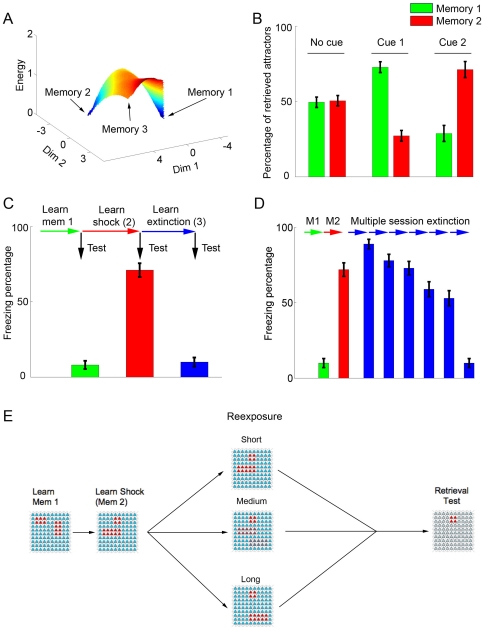
Normal learning and extinction in the model. (**A**) Energy landscape showing the relative basins of attraction after learning of memories 1 and 2 (see Methods). The learned patterns are seen as energy minima in blue, while no basin of attraction is observed for the point corresponding to memory 3. (**B**) Attractor retrieval after learning of memories 1 and 2. Upon random activation of the network (no cue), retrieval of both patterns occurs with similar probabilities, while presentation of a cue involving weak (*I* = 0.1) activation of 4 neurons pertaining to either pattern leads to preferential retrieval of this pattern. Bars represent mean ± S.E.M. of percentages of retrieved attractors over 10 sets of 100 simulations. (**C**) Fear conditioning and extinction. Memories 1, 2 and 3 are learned sequentially, with bars showing freezing percentages (mean ± S.E.M. of 100 simulations) in retrieval tests. After learning of memory 1 (green bar), little freezing occurs. Freezing increases after learning of memory 2 (red bar), but decreases again (blue bar) after extinction learning (corresponding to a single reexposure session with *t* = 10). (**D**) Extinction over multiple sessions. Learning of memories 1 and 2 occurs as in (C) (green and red bars). Extinction learning occurs through 6 reexposure sessions of intermediate duration (*t* = 6), leading to a decrease in freezing behavior in retrieval tests performed after each session (blue bars). Time-related decay (γ) occurs only before the first extinction session to allow comparison with the single-session protocol. (**E**) General protocol used to model nonreinforced contextual reexposure. Learning of memories 1 and 2 is followed by a nonreinforced reexposure session, represented by a cue pattern which varies according to reexposure duration. Memory retrieval is then tested by presentation of the context.

Figure 2 shows normal learning in the model. We first present the network with two orthogonal patterns with no overlapping active neurons, one at a time: pattern 1 (an unrelated memory) and pattern 2 (the shock memory). Presentation of these patterns leads to the formation of local energy minima corresponding to the two memories (Figure 2A). Retrieval of either one can occur upon random network initialization, while presentation of a partial cue for either of the two patterns biases retrieval towards the corresponding attractor (Figure 2B). Although we perform our simulations using only 3 patterns in a small network of 100 neurons, our network framework is capable of storing larger numbers of memories, with the absolute capacity depending on parameters such as network size and on the number of active neurons in each memory pattern, as has been shown to be the case for other attractor-based models [38], [39]. Estimations of storage capacities for different network sizes and sparseness values are shown in Figure S2, demonstrating that the model can store a reasonable number of memories, provided the number of neurons is large enough and memory patterns are reasonably sparse.

Similarly to what occurs behaviorally, extinction in the model (represented as learning of pattern 3) can occur either in a single retrieval session with a cue similar to pattern 3 (i.e. a high *t* value, representing a long retrieval session) (Figure 2C) or in multiple retrieval sessions with intermediate cues (representing multiple short sessions in which pattern 2 and 3 are both reflected in the cue) (Figure 2D). Extinction over multiple sessions occurs due to gradual weakening of the shock attractor, which is repeatedly retrieved in the presence of mismatch and thus undergoes degradation, allowing learning of a new attractor (the extinction memory) to occur eventually. This is in contrast with single session extinction, in which prompt learning of the extinction memory prevents retrieval of the original attractor and weakening of the shock representation (see Figure S3).

The sequence of patterns used to model learning followed by nonreinforced reexposure to the context, which will be used throughout the simulations concerning the effects of anisomycin, is shown in Figure 2E. Learning of patterns 1 and 2 is followed by a nonreinforced reexposure session of variable duration (modeled by changing the value of *t*), and retrieval is later measured through presentation of the context cue.

### Effects of anisomycin on different reexposure protocols

**Figure 3:**
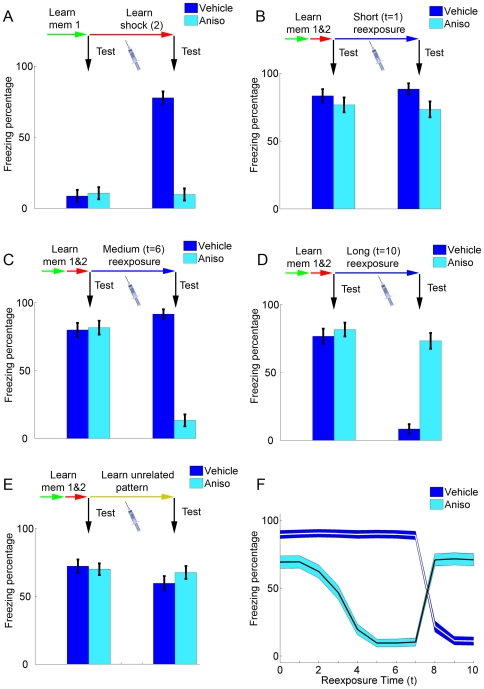
Effects of anisomycin administration in initial learning and reexposure. (**A**) Effect of anisomycin in initial learning. Memories 1 and 2 are learned sequentially, and bars represent freezing percentages (mean ± S.E.M. of 100 simulations) before (left set of bars) and after (right set of bars) learning of memory 2. Administration of vehicle (*S* = 0.8, dark blue) or anisomycin (*S* = 0, light blue) occurs in the learning session, as indicated by the syringe. Freezing increases in the vehicle group, but remains at baseline values in the anisomycin group, as learning of fear conditioning is blocked. (**B**) Effect of anisomycin in a short reexposure session. Memories 1 and 2 are learned sequentially in normal conditions (*S* = 0.8), and the left set of bars shows retrieval after learning of memory 2. During a short reexposure session (*t* = 1), vehicle (*S* = 0.8, dark blue) or anisomycin (*S* = 0, light blue) is administered, and a retrieval test after this session (right set of bars) shows that freezing remains high in both groups. (**C**) Effect of anisomycin in a reexposure session of intermediate duration. Learning sessions are performed as in (B), but with *t* = 6. Freezing remains high after the reexposure session in the vehicle group, but decreases markedly in the anisomycin group, demonstrating reconsolidation blockade. (**D**) Effect of anisomycin in a long reexposure session. Learning sessions are performed as in (B) and (C), but with *t* = 10. Freezing decreases in the vehicle group due to extinction learning, but remains high in the anisomycin group due to blockade of extinction. (**E**) Effect of anisomycin in the absence of contextual reexposure. Learning of memories 1 and 2 occurs as in (B)–(D), but the reexposure session is replaced by the learning of an unrelated pattern. Freezing in both groups remains high, demonstrating the lack of effect of anisomycin in the absence of contextual reexposure. (**F**) Summary of the effect of anisomycin in reexposure sessions of various durations. The *x* axis represents reexposure duration, while lines and blue contours show freezing percentages (mean ± S.E.M, represented in the *y* axis) of anisomycin and vehicle groups in retrieval tests performed after reexposure.

Figure 3 shows the effects of anisomycin administration (i.e. setting *S* to 0) in different learning and reexposure protocols. During initial learning, blockade of protein synthesis inhibits Hebbian modifications and prevents formation of the shock memory (Figure 3A), a finding which is consistent with the effect of anisomycin in various behavioral paradigms of learning, including fear conditioning [40].

In Figures 3B to 3E, learning of the shock memory occurs normally (*S* = 0.8), and anisomycin administration is modeled in various nonreinforced reexposure protocols with different contextual cues (see Figures 1C and 2E). In very short reexposure trials, in which the shock memory is retrieved over the full course of the retrieval session and dominates the contextual representation (i.e. low *t* values), anisomycin will have little effect on subsequent retrieval of that memory, as the degree of mismatch-induced degradation will be small even in the absence of protein synthesis (Figure 3B). This is compatible with the “simple retrieval” condition observed with short reexposure durations in experimental studies [8], [16], [41].

In reexposure trials with intermediate durations (i.e. “reconsolidation” conditions), inhibition of protein synthesis starts to exert a significant amnestic effect on subsequent retrieval trials (Figure 3C), as Hebbian learning is blocked and cannot compensate for mismatch-induced degradation of the shock memory. This effect is analogous to the reconsolidation blockade effect described in various experimental studies [3], [4]. Finally, in long reexposure trials, in which the cue pattern will be distinct enough from pattern 2 to prevent its retrieval, extinction (i.e. formation of a new attractor representing pattern 3) will occur after the reexposure session in control conditions. The burning of a new attractor in the network will also prevent mismatch degradation of the shock representation; in this case, therefore, anisomycin will block formation of the extinction memory, but will not affect the existing shock attractor, leading to preservation of the shock memory in treated animals (Figure 3D). Such results closely match the effects of reexposure time on reconsolidation and extinction found in experimental studies [8], [16], [41], [42].

In agreement with all experimental studies of reconsolidation, anisomycin administered in the absence of the original learning context for the shock memory (i.e. upon reexposure to an unrelated cue) will have no effect on its subsequent retrieval in our model, demonstrating the context-specificity of the reconsolidation blockade effect [43] (Figure 3E). The effect of reexposure duration in control conditions and in anisomycin-treated animals upon subsequent memory retrieval is summarized in Figure 3F. One can observe that the amnestic effect of anisomycin increases along with reexposure duration until the minimum duration required for extinction to occur in controls is reached (around *t* = 8). In longer reexposure conditions, on the other hand, freezing decreases in controls with increasing reexposure duration due to extinction, while anisomycin preserves the original memory by preventing extinction learning.

### Reconsolidation and extinction after different strengths of training

**Figure 4:**
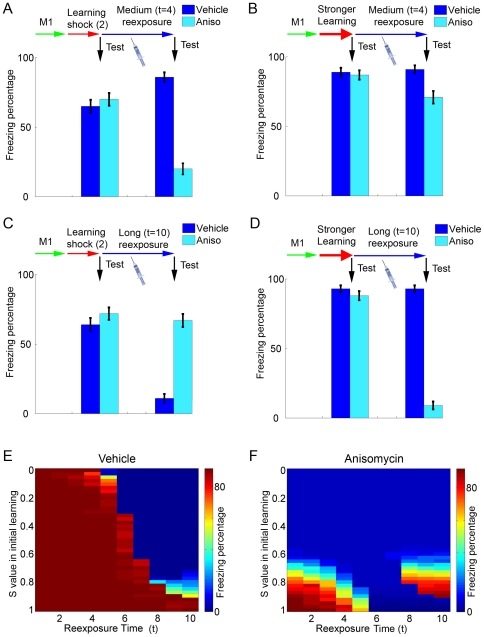
Reconsolidation and extinction after different strengths of training. (**A**) Effect of anisomycin in a reexposure session of intermediate duration after regular training. Learning sessions are performed as in Figures 3B–3E, with *S* = 0.8. Bars indicate freezing in vehicle (dark blue) and anisomycin (light blue) groups before (left set) and after (right set) reexposure with *t* = 4 (mean ± S.E.M. of 100 simulations). As occurs in Figure 3C, freezing is decreased by anisomycin administration, indicating reconsolidation blockade. (**B**) Effect of anisomycin in a reexposure session of intermediate duration after strong training. Learning and reexposure sessions are performed as in (A) except that memory 2 is strengthened by setting *S* to 0.95 during the learning session. The same reexposure protocol used in (A) causes a much lower decrease of freezing in the anisomycin group, indicating that stronger learning can protect the shock memory from reconsolidation blockade. (**C**) Effect of anisomycin in a long reexposure session after regular training. Learning sessions are performed as in (A), with *S* = 0.8. As in Figure 3D, freezing decreases in the vehicle group after reexposure with *t* = 10 due to extinction, but remains high in the anisomycin group due to extinction blockade. (**D**) Effect of anisomycin in a long reexposure session after strong training. Learning and reexposure sessions are performed as in (C), except that memory 2 is strengthened by setting *S* to 0.95 during the learning session. The same reexposure protocol used in (C) is now insufficient to cause extinction in controls, while a decrease in freezing indicating reconsolidation blockade is observed in the anisomycin group. (**E**) Color plot summarizing the effect of reexposure on freezing behavior in the vehicle group after various strengths of initial learning. Freezing percentages after a reexposure session with *S* = 0.8 (color scale) are indicated for various reexposure durations (*x* axis) with different values of *S* used in initial learning (*y* axis). Extinction (blue regions indicating low freezing) occurs at long reexposure durations, but becomes increasingly hard to induce with stronger initial learning. (**F**) Color plot summarizing the effect of reexposure on freezing behavior in the anisomycin group after various strengths of initial learning. Freezing percentages after a reexposure session with *S* = 0 (color scale) are indicated for various reexposure durations (*x* axis) with different values of *S* used in initial learning (*y* axis). Increased strengths of initial training favor maintenance of freezing (red regions) instead of reconsolidation blockade (blue regions) in short reexposure sessions, but can favor reconsolidation blockade in long reexposure sessions by preventing extinction.

As observed experimentally [7], [8], [44], the protocols necessary to induce reconsolidation and extinction in our model vary according to the strength of the original learning. In some reexposure conditions which normally induce reconsolidation in controls (and amnesia in anisomycin treated animals) (Figure 4A), anisomycin will have no effect if the initial learning of the shock memory is made stronger by increasing *S* during the training session (Figure 4B), as the stronger memory will not be as affected by the degradation caused by reexposure. Such results are in accordance with the behavioral data indicating that longer reexposure trials are needed to induce reconsolidation of stronger or more consolidated memories [8]. Another consequence of strengthening the shock memory is that longer durations of reexposure, which normally yield extinction (Figure 4C), will lead to reconsolidation instead (Figure 4D). In this case, anisomycin will not lead to memory preservation (as occurred after normal training conditions) but to reconsolidation blockade and amnesia, similarly to what has been described experimentally [7]. The effect of reexposure duration on retrieval of the shock memory for various strengths of initial learning is summarized in Figures 4E (control conditions) and 4F (anisomycin treatment during reexposure).

### Effect of memory-enhancing drugs on different reexposure protocols

**Figure 5:**
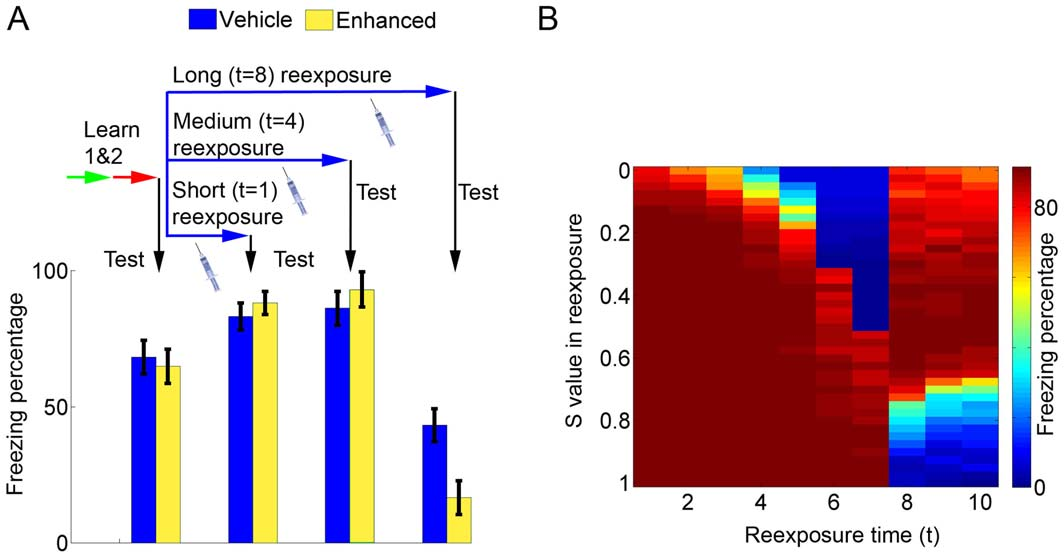
Effects of enhanced plasticity during the reexposure session on reconsolidation and extinction. (**A**) Effects of memory-enhancing drugs on reconsolidation and extinction. Learning of memories 1 and 2 occurs normally with *S* = 0.8, followed by a reexposure session of variable duration. Bars (mean ± S.E.M. of 100 simulations) indicate freezing after learning of memories 1 and 2 (first set) and after reexposure sessions of various durations in animals treated with vehicle (*S* = 0.8, blue) or a memory-enhancing drug (*S* = 0.95, yellow) in the reexposure session. Freezing is slightly increased by memory enhancement in short and intermediate reexposure sessions due to reinforcement of the shock memory. In long reexposure sessions, meanwhile, memory enhancement improves extinction and decreases freezing. (**B**) Color plot summarizing the effect of reexposure with various durations and *S* values on freezing behavior. Freezing percentages (color scale) after reexposure sessions with different durations (*x* axis) and *S* values (*y* axis) are indicated. For *t* values of up to 7, decreasing *S* below the usual value of 0.8 decreases freezing due to reconsolidation blockade, while increasing *S* values cause either no effect or slight enhancement of freezing. For *t* values of 8 or more, decreasing *S* causes preservation of freezing by blocking extinction, while increasing *S* decreases freezing by enhancing extinction.

Experimental data suggests that administration of memory-enhancing drugs such as D-cycloserine (a partial agonist of the coactivator site at the NR1 subunit of the NMDA receptor) during contextual reexposure can improve either reconsolidation or extinction, leading to an effect which is the opposite of that of anisomycin [42]. We have simulated that by increasing the value of *S* during the reexposure session, based on the enhancing effect of such drugs upon synaptic plasticity [45]. As found experimentally with D-cycloserine [42] and protein kinase A (PKA) activation [46], stimulating Hebbian plasticity during reexposure in reconsolidation conditions (*t* = 4) slightly improves subsequent retrieval of the shock memory (Figure 5A, third set of bars). This improvement was small in our simulations due to a ceiling effect, as memory in controls already approached saturation values after normal reconsolidation. On the other hand, increasing *S* during extinction conditions (*t* = 8) improves extinction and lowers subsequent fear memory retrieval (Figure 5A, fourth set of bars). These trends hold true for a range of parameters, as shown in Figure 5B, which summarizes the effects of increasing or decreasing *S* during reexposure sessions of different durations.

### Effects of blocking mismatch-induced degradation

**Figure 6:**
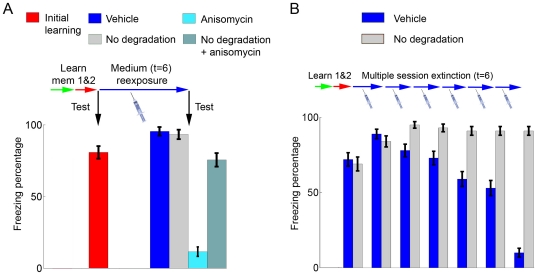
Effects of blocking degradation on reconsolidation and extinction. (**A**) Effects of blocking degradation on reconsolidation and reconsolidation blockade. Bars (mean ± S.E.M. of 100 simulations) indicate freezing percentages after initial learning with *S* = 0.8 (red) and after an intermediate reexposure session (*t* = 6) with vehicle (*S* = 0.8, *D* = 1.25, dark blue), anisomycin (*S* = 0, *D* = 1.25, light blue), degradation blockade (*S* = 0.8, *D* = 0, light gray) or anisomycin+degradation blockade (*S* = 0, *D* = 0, dark gray). Freezing decreases after anisomycin administration in reexposure as previously shown; degradation blockade has no effect on freezing on its own, but reverses the effect of anisomycin when both treatments are administered together. (**B**) Effects of blocking degradation on multiple session extinction. After learning of memories 1 and 2, multiple session extinction is induced in the vehicle group (blue bars) by performing 6 reexposure sessions with *t* = 6 as in Figure 2D. Blocking degradation by setting *D* to 0 (light gray bars) blocks the decrease in freezing, demonstrating that multiple session extinction depends on mismatch-induced degradation.

Experimental evidence for the effects of blocking protein degradation on memory (usually achieved through the use of inhibitors of the ubiquitin-proteasome cascade) is somewhat controversial, with different effects described on initial learning [36], [47], [48], [49] and reconsolidation [35], [36], [47]. It has recently been suggested, however, that protein degradation is necessary for the amnestic effect of anisomycin on reconsolidation to occur [35], [36]. This indeed occurs by blocking mismatch-induced degradation (i.e. setting *D* to 0) in our model, which does not affect memory reconsolidation by itself, but prevents the effect of anisomycin on subsequent retrieval (Figure 6A). Blocking mismatch-induced degradation will also prevent multiple session extinction (Figure 6B), as shown experimentally in one of these studies [36]. This result demonstrates that the mismatch-induced degradation system has a physiologic role in our model, as it allows nonreinforced trials of intermediate duration to lead to extinction when performed repeatedly, as opposed to the reinforcement of the original memory which occurs in the absence of degradation. When compared to experimental findings, it also suggests that protein degradation through the ubiquitin-proteasome system could be one of the mechanisms involved in mismatch-induced degradation of synaptic changes.

## Discussion

The results presented show that our attractor network-based model accounts for the main experimental results concerning the effects of anisomycin on reconsolidation and extinction of fear conditioning in different reexposure protocols. More specifically, the model is in agreement with experimental data suggesting that nonreinforced contextual reexposure has three possible outcomes, namely: (a) simple retrieval (i.e. absence of reconsolidation or extinction), in which anisomycin has no effect on memory; (b) reconsolidation, in which anisomycin causes amnesia due to blockade of this process; and (c) extinction, in which the behavioral response to the original memory is reduced in controls, but preserved in animals treated with anisomycin [8], [16], [41].

There are three main assumptions of the model that allow such results to be obtained. The first one is the existence of attractor dynamics in the brain, which produces nonlinear transitions between the retrieval of an established attractor and the instatement of a new attractor in the network as a function of the perceived contextual representation during reexposure. The second one is the existence of a protein-synthesis independent system which acts to counteract Hebbian plastic changes in synapses in response to mismatch between an established memory and new information. The third assumption is that the degree of dissimilarity between an animal's contextual representation upon nonreinforced reexposure and the representation of the original learning will increase with longer durations of reexposure.

Concerning this last point, it should be noted that the data presented can also be interpreted with *t* assumed to represent the degree of dissimilarity between the reexposure session and the original learning trial, rather than reexposure duration. In this case, our results indicate that reconsolidation in our model can be induced by an experience which is similar to that of the original learning, but not by one which is identical to it (in which no mismatch will occur) or radically different from it (which will lead to instatement of a new attractor and also prevent mismatch). This is in agreement with experimental data concerning the need of both similarities (e.g. a similar environment) and differences (e.g. absence of shock, differences in objects or platform location) between the original learning trial and the reexposure trial for reconsolidation to occur [14], [15], [50], [51]. It is also in line with the view that either updating of an existing memory trace or formation of a new one can occur upon reexposure, depending on the degree of contextual similarity [52].

It is noteworthy that the simple retrieval condition described in our model provides an interesting framework to interpret studies which failed to demonstrate reconsolidation, but used reexposure trials in which duration was short [9] or mismatch was not a prominent feature [10], as these might have been insufficient to induce significant updating of an existing attractor. The model also accounts for the fact that different reexposure protocols are required to induce reconsolidation and extinction of memories of different strengths [7], [8], which could furthermore explain the different susceptibility of recent and remote memories to these processes [3], [8], [53] if one assumes that reinforcement of memory traces occurs over time [31]. This assumption can also reconcile our model with results showing that amnesia induced by reconsolidation blockade can be transient [54], [55], as synaptic reinforcement could lead to regeneration of the trace in the hippocampus or neocortex [30]. Finally, if one assumes that mismatch-induced synaptic changes involve protein degradation, the model can explain the recently described dependence of reconsolidation blockade on the activity of the ubiquitin-proteasome system [35], [36].

One should keep in mind that the dichotomy between retrieval-induced, Hebbian plasticity based on protein synthesis and mismatch-induced synaptic changes based on protein degradation is an obvious biochemical simplification, and that both the *HLP* and *MID* terms of the model certainly involve more than protein synthesis and degradation in a biological setting. In fact, it is likely that any plastic change in synapses involves both synthesis and degradation of specific proteins, which would account for studies showing the requirement of the ubiquitin-proteasome cascade for initial learning [47], [48], [49] and normal reconsolidation [47] to occur. It is also possible that both processes share other mechanisms such as a dependence on NMDA receptors, as blockade of these receptors has been shown either to induce [42], [56], [57] or prevent [58] reconsolidation blockade depending on the study. Nevertheless, our simplification is probably valid to account for the experimental results we have tried to model, if one assumes that protein synthesis blockade will *preferentially* affect mechanisms underlying *HLP*, while degradation blockade will *preferentially* have an effect on *MID*, at least under some experimental conditions.

While *HLP* seems readily relatable to Hebbian-like plasticity mechanisms such as long-term potentiation (LTP), the mechanisms underlying mismatch detection and *MID* in the model remain an open question; nevertheless, it seems natural to speculate possible relationships of this process with long-term depression (LTD) and synaptic depotentiation phenomena. LTD has been shown to involve the ubiquitin-mediated degradation of proteins such as PSD-95 [59] and, although it normally requires protein synthesis [60], this requirement is not observed for all of its forms [61]. Protein synthesis is also not required when low-frequency stimulation is used to reverse preexisting LTP [62]; this phenomenon, usually known as depotentiation, shares several features with LTD [63], but is usually restricted to a short time window (i.e. hours) following induction of LTP. However, since *MID* in our model happens in many synapses which also exhibit Hebbian plasticity during reexposure, it is possible that LTP-mediated changes happening during reexposure could reinstate the lability of synaptic weights to protein synthesis-independent depotentiation.

If *MID* can indeed be related to LTD-like phenomena, then changes in firing rates and spike synchronization among coactive neurons during reexposure could mediate mismatch detection through spike-time-dependent plasticity (STDP) [64], [65], which has recently been shown to be compatible with firing-rate based models of LTP and LTD [66] and with autoassociative plasticity in recurrent networks [28], [67]. In this case, if cue currents to shock and context neurons are unambiguous and synchronized (i.e. simple retrieval), such neurons should spike at high firing rates and in close temporal proximity, leading to the development of LTP in their mutual connections. On the other hand, if cue inputs to context and shock neurons become more distinct (i.e. reconsolidation), firing rates and synchrony will decrease and LTD should become more prominent. Thus, if LTP is disrupted by protein synthesis inhibition, this could conceivably lead to reconsolidation blockade. However, if reexposure is long enough to allow extinction, silencing of the shock neurons would prevent the occurrence of either LTP or LTD, as both depend on neuronal spiking; thus, the disruptive effect of blocking protein synthesis on the shock memory would not occur in extinction conditions, as observed in our simulations.

The central core of our model's results is accounted for by the attractor properties of the Hopfield network, which allow it to perform either pattern completion (leading to retrieval of the shock memory upon presentation of the context alone) or pattern separation (leading to the nonlinear transition between retrieval of an established attractor and the formation of a new one). Attractor networks have been classically thought to exist in the hippocampus, particularly in CA3 [25], [68], and both pattern completion and separation have been suggested to occur in the hippocampal formation by theoretical models [25], [69] and behavioral findings [70], [71], [72]. Moreover, electrophysiological evidence from place field recordings suggests the occurrence of discontinuous attractor transitions in the hippocampus [19]. If our attractor network is thought to represent the CA3 region, an interesting finding is that our results depend on a limited degree of overlap between the shock and extinction patterns (7 to 28%, as observed in Figure S4), which is in the range observed for place field representations of similar environments in CA3 [73]; if overlap is higher, pattern completion will prevent extinction learning. This is in line with the view that some degree of pattern orthogonalization by the dentate gyrus is necessary to allow CA3 to separate information between similar contexts [74] – or, in the case of extinction, between different representations of the same context.

Nevertheless, the existence of attractor dynamics in other structures as well has also been suggested by experimental evidence, such as the sustained activity of neocortical regions after removal of sensory stimuli [21], [75], the observation of abrupt pattern transitions in response to gradually changing stimuli in the olfactory bulb [22], and the recent electrophysiological demonstrations of recurrent connectivity in the lateral amygdala [76]. Therefore, although we have developed our model to describe phenomena thought to occur in the hippocampus, such as contextual recognition, it is not dependent on specific features of hippocampal anatomy (i.e. all it requires anatomically is the existence of recurrent connections which can sustain attractor properties). Thus, the mechanisms proposed by the model could conceivably happen in other structures as well, as most of the evidence showing the effects of reexposure duration on reconsolidation and extinction comes from studies using systemic injections [8], [14], [16], [41], [42].

The amygdala in particular has been shown to be involved in the reconsolidation of auditory fear conditioning [4], and pharmacological transitions from reconsolidation to extinction according to reexposure duration have been observed with intra-amygdala injections [42], [77]. Therefore, it is possible that recurrent connectivity in this structure [76] could support local attractor functioning, as proposed by other authors [78]. The observation of reconsolidation and extinction in invertebrates such as crabs [79] and fruitflies [41] also suggests that network effects mediating these processes can exist in the absence of a hippocampus-like structure; in this sense, our demonstration that a simple network with recurrent connections can mediate attractor properties subserving reconsolidation and extinction could be significant for interpreting these data.

Still, although these systemic interactions mediating reconsolidation and extinction are far from clear, and probably involve complex modulations among structures [44], we propose that the existence of attractor dynamics in the hippocampus could play a central role in determining the dominant process induced by contextual reexposure, at least in context-dependent and spatial memory tasks. The bidirectional communication of this structure with the entorhinal cortex could provide an anatomical substrate for the feedback loop between online contextual representations (mediated by the cortex) and the memory network (located in the hippocampus) proposed in our framework. In particular, the CA1 area has been proposed to be responsible for comparing current contextual representations (entorhinal cortex inputs) and stored attractors (CA3 inputs) [80], [81], and could be involved in detecting mismatch between these representations. This hypothesis is further strengthened by the recent demonstration that reexposure to a learning context can induce transient weakening of LTP-related changes in CA3-CA1 synapses [82]. In Figure S5 and in the Supporting Text S1, we propose a simple model for how mismatch detection by the CA1 region could mediate reconsolidation and account for these results. In this model, differences in activity between the CA3 and CA1 regions lead to mismatch-induced degradation and reconsolidation phenomena, while transitions between reconsolidation and extinction are accounted for by the attractor dynamics in the CA3 region.

Still regarding the CA1 area, an interesting analogy can be drawn between our computational results, based on fear conditioning experiments, and electrophysiological data derived from hippocampal place cell recordings in this region. Different studies have suggested that “morphing” between two contexts with different place fields can either lead to continuous updating of these place fields [83] or to abrupt changes from one place field representation to another [19], probably depending on the protocol through which intermediate patterns are presented [29]. These findings have been suggested to be related to attractor dynamics [19], [29] and show some similarities with the results of our model, in which fear-related attractors can be either “updated” through reconsolidation or replaced with a new attractor encoding extinction. The fact that fear conditioning can induce partial remapping of place fields for the conditioning context [84] suggests that such an analogy between shock/non-shock representations of a context and different place field representations may be warranted.

As expected from a theoretical model, our framework generates a number of predictions concerning the effects of amnestic agents on different learning and reexposure protocols. Some simple experimental predictions on the behavioral, biochemical and electrophysiological levels which would lend support to the general framework of the model are the following:

- Only an attractor which is actively retrieved by the network should be affected by reconsolidation blockade, as mismatch-induced degradation will act upon the connections that maintain this attractor. Such specificity has already been shown for memories learned in different contexts [43] and our data predict that it can also happen for different representations of the same environment. This suggests that the extinction memory itself could be subject to reconsolidation once it becomes dominant over the original memory, if some form of mismatch (e.g. a reminder of the shock memory) is introduced in a reexposure session (for an example of this, see Figure S6). The first experimental evidence for this prediction has recently been reported using the inhibitory avoidance task [85].
- If the effects we describe are indeed happening in the hippocampus, one would expect them to be observed not only in contextual fear conditioning, but also in spatial memory paradigms. Therefore, in spatial tasks in which more than one behavioral strategy can be learned, such as reversal learning protocols in the Morris water maze in which two distinct platform locations are learned in sequence, our model predicts that the one which is retrieved by an animal at the time of nonreinforced reexposure should be the one subject to reconsolidation blockade. Besides providing support for the model's general framework, such a finding would strengthen the case for involvement of the hippocampus in mediating reconsolidation/extinction transitions.
- The mismatch degradation system described in our model should be activated more strongly after reconsolidation-inducing reexposure protocols than after extinction-inducing ones. This leads to the prediction that molecular cascades involved in this system should be activated differently after short and long reexposure durations, and that signatures of this differential activation could be detected through molecular/biochemical analysis of brain tissue. Interesting candidates to be evaluated for this purpose include the ubiquitin-proteasome system [36], and possibly the endocannabinoid system, which has shown to modulate reconsolidation and extinction in opposite ways [86].
- If one assumes the analogy between shock/non-shock representations and place field representations as valid, this means that extinction-inducing protocols should lead to a partial remapping of place cells in the conditioning context, similar to the one observed with initial conditioning [84]. This more indirect prediction is based on the assumption that place field representations can also be stored as attractors, as suggested by electrophysiological data [19].
- If the abovementioned prediction is proved true, an additional electrophysiological prediction is that the time for place cell remapping during fear extinction should match the time course of the transition between reconsolidation and extinction in the behavioral protocol used (which has usually been shown to be in the range of 5–30 minutes in studies of contextual fear conditioning tasks in rodents [8], [16]).

Finally, although our model argues for a network view of reconsolidation and extinction, this does not mean that differences between the two processes do not exist at the biochemical level. On the contrary, it is likely that dissimilarities between them also depend on the activation of different molecular cascades, as suggested by some studies which have pointed out pharmacological and biochemical differences between the two processes [8], [86], [87], [88]. In this sense, our model provides at least one explanation why some drugs could have differential effects on reconsolidation and extinction – namely, that they could be targeting mechanisms which are not involved in classical Hebbian plasticity, but rather in trace labilization (i.e. affecting *MID* but not *HLP* in our model). If this is the case, the same drug could produce differential effects in reconsolidation and extinction trials under some conditions (see Figure S7), as has been recently shown with drugs acting on the CB1 receptor [86]. Naturally, it is also possible that there are other instances of memory modulation that were not included in our model and could account for these effects.

In summary, by assuming the existence of attractor dynamics and mismatch-induced updating of plastic changes in neural networks, we provide a parsimonious explanation for the occurrence of reconsolidation and extinction after nonreinforced reexposure in fear conditioning tasks. Although in a biological setting the modulation of these processes probably involves many other factors as well, we believe our model is an interesting proof of principle of the fact that both reconsolidation and extinction can be explained by a unified set of plasticity mechanisms (i.e. the *HLP* and *MID* terms in our equations), albeit operating in different synapses. Therefore, the usual tenet that reconsolidation and extinction represent distinct processes at the cellular and molecular level might not be entirely true, as differences between the network aspects of the two processes could be more important in their distinction. This view is supported by the striking similarities between the pharmacology of reconsolidation and that of extinction, which certainly outnumber their dissimilarities in the existing literature. Such aspects should be taken into account for adequately translating knowledge from animal studies of memory into useful clinical approaches for the treatment of psychiatric disorders.

## Methods

### General model framework

In line with previous research, we model the attractor network responsible for storing the memory patterns as a fully connected neural network [24], [29], [30], [31]. Neuronal activities in this network are determined by Eq. (1), which fully defines its dynamics and constrains neural activation (*u*) to values between 0 and 1 through the term ½(1*+tanh*(Σ*w_ij_u_j_ + I_j_*). This represents a change from the original Hopfield formulation, in which *u* is unbounded and can achieve negative values as well. In that formulation, however, *u* is typically regarded as the membrane potential, while *V(u) = (tanh(u)+1)/2* would represent the firing rate or activity level of a neuron. In this sense, in our model *u* can be thought of as a direct measure of the firing rate, without the intermediate step of calculating the membrane potential.

As mentioned in the results session, the 0/1 implementation can reflect the firing rate and connectivity of neurons in a more realistic way, as it does not assume unrealistic features such as symmetric connectivity and reinforcement between silent neurons; this kind of change from the original Hopfield formulation has also been implemented by other authors in different ways [89], [90]. Furthermore, the use of a 0/1 activation scheme also prevents the retrieval of “mirror attractors” (i.e. retrieval of a pattern diametrically opposite to the one which was originally learned) and diminishes the retrieval of spurious patterns when sequences of correlated patterns are learned, as in the case of our simulations, since it prevents the strengthening of connection between inactive neurons, which can lead to the development of abnormal connectivity between neuronal populations when the patterns used are not completely arbitrary.

### Modeling of synaptic weight strengthening and degradation

The final steady state pattern achieved by Eq. (1) will in turn induce changes in the synaptic weight matrix 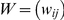 as determined by Eq. (2), with the *HLP* and *MID* terms defined in equations (3) and (4), respectively. In these equations, the notation B*A denotes connections from *A* to *B*. Therefore, note that the first term in Eq. (3) represents the strengthening of positive synaptic connections among coactive neurons, while the second term models a reduction in the strength of synaptic connectivity from active to inactive neurons. Moreover, as the entries of 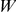 can achieve negative values, the second term in (3) can bring about inhibition from active to inactive neurons, as in classical formulations (see Figure 1D). To prevent a memory or a set of memories from completely dominating and suppressing the other memories, we require that the magnitude of synaptic entries in the matrix 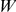 saturates at a maximum value s_0_. We implement this by truncating the entries that become too large back to s_0_, and by using a similar procedure for synaptic values that decrease below −s_0_.

After reaching the steady state upon a cue presentation, all units belong to one of four categories: *AA*, *SA*, *AS* and *SS*, where *A* stands for *Active* and *S* stands for *Suppressed*, with the first letter indicating the nature of the cue currents and the second letter denoting the final unit activity upon reaching the steady state. For the computation of the mismatch vector, we normalize the cue currents 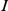, making them achieve values between 0 and 1 through a transformation defined by 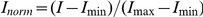. Therefore, it follows from Eq. (4) that when the retrieved pattern is in complete agreement with the input cue, all entries in the matrix *MID* have zero values, since all units belong either to category *AA* or to category *SS*.

When mismatch occurs between the attractor network pattern and the cue currents, this means that there are units pertaining to either *AS* or *SA* categories – that is, there are neurons that were suppressed in the retrieved pattern despite activation by the cue current and, conversely, neurons that were active despite cue suppression. The synaptic changes induced by mismatch occur only at the connections linking: (a) active units (*AA* or *SA*) to *AS*, and (b) active units to *SA*. As a result, in the first case, mismatch-induced degradation acts to decrease the inhibition from active units towards units that are rendered inactive despite the existence of excitatory cue currents arriving at these neurons (*AS* units; see Figure 1E). Thus, upon subsequent presentation of the same cue pattern, the overall drive (i.e., cue currents plus within network influences) to the *AS* units is increased, making these units more likely to switch to the *AA* category. Similarly, in the second case, the strength of connections from active units to *SA* units decays to lower values as a result of the mismatch-induced degradation (Figure 1E). Consequently, *SA* units become more likely to switch to the *SS* category upon subsequent presentation of the same pattern. Note that the effects mentioned here are a consequence of the definition of the mismatch vector (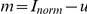), upon which *MID* is dependent (Eq. (4)). For instance, an *AS* unit *j* has its correspondent *m* entry value equal to *m_j_* = +1−0 = +1, whereas a *SA* unit *k* has *m_k_* = 0−1 = −1. Therefore, by employing 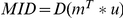, the connection strength from an active unit *i* (i.e, *u_i_* = +1) to an *AS* unit *j* will be changed by *MID_ji_* = +*D*, whereas the connection to a *SA* unit *k* will change by *MID_ki_ = −D*. Taken together, these two types of mismatch-induced degradation facilitate the learning of new memory patterns that introduce conflicting information.

### Modeling of retrieval tests and nonreinforced reexposure

Memory retrieval is tested by presenting the cue pattern which represents the context, with 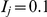 of its strength at training for context neurons *j* and 0 for other neurons (Figure 1B), and observing the attractor to which the network evolves. In order to have a closer correlation between attractor retrieval in our computational model and the behavioral measures of memory used in experimental studies of fear conditioning, we model the retrieval of a particular memory pattern as leading to a certain amount of freezing during the test session. Therefore, we assume that upon retrieval of the shock pattern the animal exhibits a high amount of freezing (90% of the test duration), while other memory patterns induce a low, baseline freezing time (10% of the test duration) (see Figure S1).

Cue inputs during the reexposure sessions are modeled by:
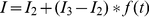
(5)where vectors 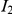 and 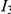 represent the cue inputs for patterns 2 and 3 (Figure 1B), respectively, and *t* represents the amount of reexposure time (which varies between minimum and maximum values *t_min_* = 0 and *t_max_* = 10 in the simulations). The function 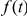 monotonically varies between 0 and 1 and determines the ratio between pattern 3 and pattern 2 present in the cue input 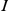, which is thus assumed to depend on the reexposure time *t*. We use a sigmoid defined by 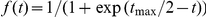, but any monotonically increasing function onto [0,1] provides qualitatively similar results.

### Visualization of attractor basins

In agreement with previous research, the strength of the stored memories could be estimated from statistics of full pattern retrieval induced by either partial cue presentation or random initialization of the neural units. In addition, we also developed a new method (used in Figure 2 and Figures S3 and S6) to estimate the basins of attraction for these patterns, defined as follows. Although each pattern constitutes a point in a large *N*-dimensional space (*N* = 100), the number of patterns *P* presented to the network is low (*P* = 3). This allowed us to use Multiple Discriminant Analysis (MDA) [91] to project these patterns into a low-dimensional encoding subspace of dimension *P−1* (in the examples used in this work the resulting dimension is *D* = 2, a plane). This projection can be obtained by performing and eigenvalue/eigenvector decomposition of the total covariance matrix *S_b_* given by the formula:
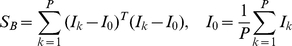
(6)Here, 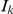 is the corresponding pattern for each class and 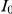 is the global mean vector. This method allows the projection of continuous *N*-dimensional neural states into this subspace, using the matrix comprised by the first *P*−1 eigenvectors. We then compute their corresponding energy (Lyapunov) function [24] in the original space, using the formula:
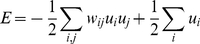
(7)


Finally, the average energy corresponding to a region in the low-dimensional space is determined as the local mean energy over a set of nearest neighbors and displayed as a 3D color map. While we do not prove that network dynamics converge to a local minimum for all possible initial states, numerical simulations indicate that this is indeed true for all cases analyzed with the 0/1 network used in our work. As a result, this method is useful for providing enhanced intuition about the relative strength of the stored patterns, allowing direct visualization of their corresponding basins of attraction.

### Model parameters

The model parameter values employed in simulations were τ = 1 and *N* = 100 and γ = 0.15; s_0_ = 1; *S* = 0.8; *D* = 1.25 (unless where otherwise noted). 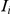 varied from −5 to +5 for each neuron according to the learning pattern presented (see Figures 1C and 2E). Although parameters such as *S* and *D* cannot be directly estimated from experimental evidence, results qualitatively similar to ours can be obtained for a reasonably wide range of parameters, albeit with reconsolidation and extinction occurring at different values of *t*, as observed in Figure S8.

For each protocol studied, we ran 100 simulations with different initial conditions of activation randomly chosen from a uniform distribution on [0,0.1], and the reported result constitutes the mean ± S.E.M. of these simulations, unless where otherwise specified.

## Supporting Information

Figure S1 **Comparison of attractor retrieval and freezing percentages.** (**A**) After learning unrelated memory 1, testing yields retrieval of this attractor (green bar, mean ± S.E.M. of 10 sets of 100 simulations) even in the presence of contextual cues, leading to a low percentage of freezing (grey bar). (**B**) After learning of memory 2, retrieval of its attractor (red bar) predominates over that of memory 1, leading to substantially higher freezing. (**C**) After a long duration of reexposure leading to extinction (*t* = 8), retrieval of attractor 3 becomes dominant (blue bar), causing decrease of freezing. (**D**) Correlation between retrieval of attractor 2 (*x* axis) and freezing percentages (*y* axis, mean ± S.E.M.) demonstrates a linear relation.
(PDF)

Figure S2 **Effect of network size and pattern sparseness on memory storage capacity.** (**A**) Storage capacity of networks with different number of neurons. Different numbers of arbitrary patterns (*x* axis) with random overlap are stored in the synaptic weight matrices W of networks of different sizes, varying from 100 to 700 neurons (color lines), using the same learning rule as in our model (Eq. (3)). Parameters are similar to those used in other simulations, including the size of the memory patterns (14 neurons) and corresponding cues (4 neurons). Retrieval is evaluated by providing the network with a cue pertaining to one of the learned memory patterns and testing the correlation between the activity of neurons in the retrieved pattern and in the original memory pattern, as done by other authors [92], [93]. For each point, 200 retrieval trials using randomly chosen cues and random initial conditions were performed, and the percentage of trials in which successful retrieval occurred (defined as an *r* value > 0.7 for the correlation) is shown. Memory capacity increases steadily with network size, showing that our network model is able to store a large amount of patterns if sufficient neurons are added to the network. (**B**) Same as in (A), except that the patterns and cues used are half the size as in (A) (i.e. 7 neurons/pattern, 2 neurons/cue). One can observe that memory capacity greatly increases using sparser patterns, as reported to be the case in previous attractor models.
(PDF)

Figure S3 **Comparison of relative memory strengths after single session and multiple session extinction.** (**A**) Basins of attraction after single session extinction. Learning of memories 1, 2 and 3 occurs as in Figure 2C, with extinction learned in a single reexposure session with *t* = 10. Energy landscape shows relative basins of attraction after learning of the 3 memories. The energy minimum for memory 2 persists, but is higher than that of memory 3, leading to behavioral dominance of the extinction memory. (**B**) Basins of attraction after multiple session extinction. Learning of memories 1 and 2 occurs as in (A), while extinction occurs through 6 reexposure sessions with *t* = 6 as in Figure 2D. The energy minimum for memory 2 is significantly reduced by multiple sessions of extinction when compared to a single session, due to the effects of mismatch-induced degradation. This could be related to the finding that spaced extinction trials can lead to less spontaneous recovery and renewal of the original memory than massed trials [94].
(PDF)

Figure S4 **Effect of pattern overlap on the development of reconsolidation and extinction.** (**A**) Freezing rates in a retrieval test performed after a reexposure session of variable duration in control conditions (*S* = 0.8) with different degrees of overlap between memory 2 and memory 3 used in the simulations. The percentage of coactive neurons in both patterns is indicated in the *y* axis, while the duration of reexposure (*t*) is indicated in the *x* axis, with the color scale representing freezing at a subsequent retrieval test. One can observe that above a certain degree of overlap (around 30% of active neurons in common), extinction does not occur with the parameters used in the simulations, suggesting that some degree of pattern separation is necessary for extinction learning. (**B**) Freezing rates after reexposure of various durations under anisomycin (*S* = 0). Blockade of *HLP* shows that reconsolidation blockade occurs in a wider range of overlap (from 7 to 80% of active neurons, approximately) than extinction blockade, which is only observed with up to 30% overlap, as in (A).
(PDF)

Figure S5 **CA3-CA1 model for mismatch detection, reconsolidation and extinction.** (**A**) Model scheme. Output from the entorhinal cortex (EC) reaches both the CA3 and CA1 regions, providing information on the current context. CA3 neurons possess autoassociative connections, and send information on their retrieved attractor to CA1. (**B**) Initial learning, reconsolidation and extinction of aversive memories in the CA3-CA1 model. Left column shows a more detailed view of the model scheme, with sample neurons representing the context (square), shock (circle) and absence of shock (triangle). Middle column shows activation of the same neurons during initial learning, reconsolidation and extinction, while the right column shows the synaptic weight changes caused by this activation. (I) Initial learning. Context and shock neurons are activated in all three networks (middle column), leading to strengthening of synapses (solid arrows) between coactive neurons (both in CA3 collaterals and in CA3-CA1 synapses) and inhibition of non-shock neurons in CA3 (circle-capped lines), as shown in the right column. (II) Reconsolidation. Ambiguous information from the EC leads to partial activation of shock and non-shock neurons in CA1, while CA3 still retrieves the original pattern (middle column). The mismatch generated between CA3 and CA1 shock neurons leads to mismatch-induced degradation of their connections (discontinuous arrow), which is compensated by Hebbian learning both within CA3 and in CA3-CA1 connections. (III) Extinction. Cue patterns indicating absence of shock lead to instatement of this pattern both in CA1 and CA3 (middle column). Synaptic weight changes show formation of a new attractor representing extinction in CA3 and strengthening of connections between non-shock neurons in CA3 and CA1, while connections between shock neurons remain unaltered. (**C**) Effect of anisomycin in reexposure sessions of various durations in the CA3-CA1 model. The *x* axis represents reexposure duration, while the lines show freezing percentages (*y* axis) of vehicle (red) and anisomycin (blue) groups in retrieval tests performed after reexposure. The dependence of retrieval on reexposure duration in both groups is qualitatively similar to what is observed with the general model in Figure 3F.
(PDF)

Figure S6 **Selectivity of reconsolidation blockade to the retrieved attractor.** (**A**) Reconsolidation of fear conditioning. Bars show freezing percentages after learning memories 1 and 2 (left) and after a subsequent reexposure session with *t* = 5 (right) in animals treated with vehicle and anisomycin in reexposure. As in Figure 3C, freezing remains high after reexposure in the vehicle group, but decreases due to reconsolidation blockade in the anisomycin group. (**B**) Reconsolidation of fear extinction. Left bars show freezing after learning of memories 1 and 2 followed by fear extinction with a long reexposure session (*t* = 10), which leads to learning of memory 3. As occurs in Figures 2C and 3C, memory 3 is dominant after this protocol, leading to low freezing. Subsequently, a reexposure session with the same duration as in (A) is performed; vehicle or anisomycin is administered in this session, and right bars show freezing in a subsequent retrieval test. Freezing remains low in the vehicle group, but increases in the anisomycin treated group, indicating that the extinction memory, which was preferentially retrieved during reexposure, becomes susceptible to reconsolidation blockade. (**C**) Energy landscapes showing relative basins of attraction for memories 1, 2 and 3 after learning of memories 1 and 2 (left) and after a subsequent reexposure session (*t* = 5) with vehicle (middle) or anisomycin (right). Right panel shows a decrease in the basin of attraction of memory 2 (shock memory). (**D**) Energy landscapes showing relative basins of attraction for memories 1, 2 and 3 after learning of memories 1, 2 and 3 (left) and after a subsequent reexposure session (*t* = 5) with vehicle (middle) or anisomycin (right). Right panel shows a decrease in the basin of attraction of memory 3 (extinction memory).
(PDF)

Figure S7 **Differential effects of enhanced mismatch-induced degradation on reconsolidation and multiple session extinction.** (**A**) After learning of memory 2, increasing *D* from 1.25 (blue bar) to 1.5 (orange bar) during a reexposure session (*t* = 7.5) leads to a decrease in freezing as compared to the vehicle group, as the reinforcement of memory 2 is impaired. (**B**) In a multiple session extinction protocol (6 sessions with *t* = 6), the same increase in *D* used in (A) leads to an acceleration of memory extinction.
(PDF)

Figure S8 **Effect of** ***S*** **and** ***D*** **variations on the development of reconsolidation and extinction.** (**A**) Freezing rates in a retrieval test performed after a reexposure session of variable duration (*x* axis) with different values of *S* (*y* axis) used throughout the simulations (i.e. learning of memories 1 and 2 and reexposure sessions), while *D* is fixed at 1.25. One can observe that, with the other parameters kept fixed, extinction happens for *S* values varying from 0.6 to 0.9, although at different reexposure durations. (**B**) Freezing rates retrieved as in (A), but with anisomycin administration (*S* = 0) simulated during reexposure, with different values of *S* used during learning of memories 1 and 2. Reconsolidation occurs for values of *S* varying from 0.7 to 1, albeit at variable reexposure durations. (**C**) Freezing rates retrieved as in (A) and (B), but with different values of *D* throughout the simulations, while *S* is fixed at 0.8. *D* values have little effect on the occurrence of extinction, as there is no mismatch between current context and retrieved attractors in extinction conditions; however, high values of *D* (i.e. above 1.6) can lead to an amnestic effect under reconsolidation conditions, even in the absence of anisomycin. (**D**) Freezing rates retrieved as in (C), but with anisomycin administration during reexposure. Reconsolidation blockade is observed at all values of *D* included in the simulations, although higher values lead to amnesia at progressively shorter reexposure durations.
(PDF)

Text S1 (PDF)
