# Supplementary material for: A Mismatch-Based Model for Memory Reconsolidation and Extinction in Attractor Networks
Source: PLoS One. 2011 Aug 3;6(8):e23113. doi: 10.1371/journal.pone.0023113 (PMC3149635; doi:10.1371/journal.pone.0023113)
Supplement: Figure S1 — Comparison of attractor retrieval and freezing percentages. (A) After learning unrelated memory 1, testing yields retrieval of this attractor (green bar, mean ± S.E.M. of 10 sets of 100 simulations) even in the presence of contextual cues, leading to a low percentage of freezing (grey bar). (B) After learning of memory 2, retrieval of its attractor (red bar) predominates over that of memory 1, leading to substantially higher freezing. (C) After a long duration of reexposure leading to extinction (t = 8), retrieval of attractor 3 becomes dominant (blue bar), causing decrease of freezing. (D) Correlation between retrieval of attractor 2 (x axis) and freezing percentages (y axis, mean ± S.E.M.) demonstrates a linear relation. (PDF) [file pone.0023113.s001.pdf]

SUPPORTING FIGURE 1

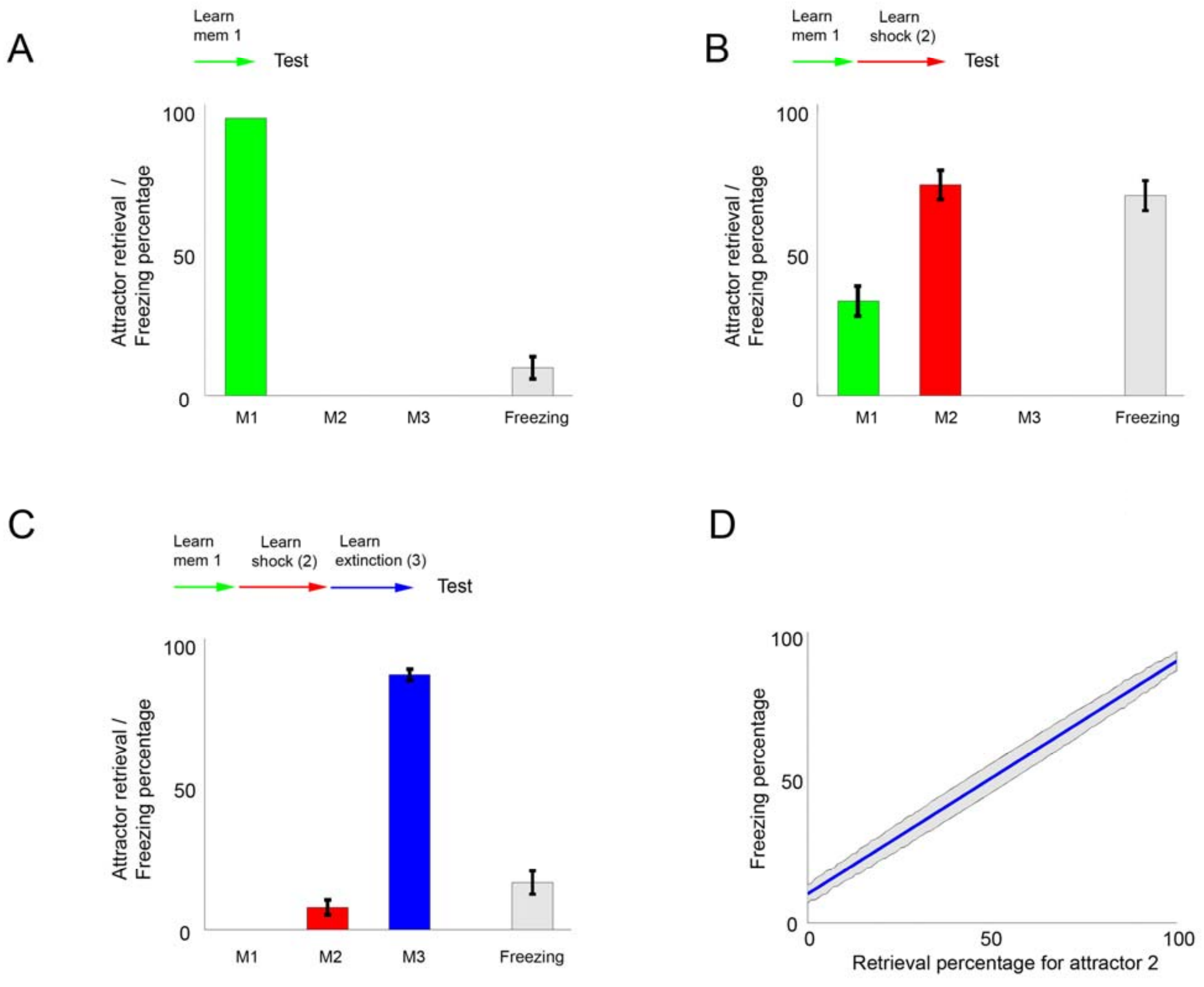

Supporting Figure 1. **Comparison of attractor retrieval and freezing percentages.** **(A)** After learning unrelated memory 1, testing yields retrieval of this attractor (green bar, mean ± S.E.M. of 10 sets of 100 simulations) even in the presence of contextual cues, leading to a low percentage of freezing (grey bar). **(B)** After learning of memory 2, retrieval of its attractor (red bar) predominates over that of memory 1, leading to substantially higher freezing. **(C)** After a long duration of reexposure leading to extinction (t = 8), retrieval of attractor 3 becomes dominant (blue bar), causing decrease of freezing. **(D)** Correlation

between retrieval of attractor 2 (*x* axis) and freezing percentages (*y* axis, mean ± S.E.M.) demonstrates a linear relation.
