# Supplementary material for: A Mismatch-Based Model for Memory Reconsolidation and Extinction in Attractor Networks
Source: PLoS One. 2011 Aug 3;6(8):e23113. doi: 10.1371/journal.pone.0023113 (PMC3149635; doi:10.1371/journal.pone.0023113)
Supplement: Figure S2 — Effect of network size and pattern sparseness on memory storage capacity. (A) Storage capacity of networks with different number of neurons. Different numbers of arbitrary patterns (x axis) with random overlap are stored in the synaptic weight matrices W of networks of different sizes, varying from 100 to 700 neurons (color lines), using the same learning rule as in our model (Eq. (3)). Parameters are similar to those used in other simulations, including the size of the memory patterns (14 neurons) and corresponding cues (4 neurons). Retrieval is evaluated by providing the network with a cue pertaining to one of the learned memory patterns and testing the correlation between the activity of neurons in the retrieved pattern and in the original memory pattern, as done by other authors [92], [93]. For each point, 200 retrieval trials using randomly chosen cues and random initial conditions were performed, and the percentage of trials in which successful retrieval occurred (defined as an r value > 0.7 for the correlation) is shown. Memory capacity increases steadily with network size, showing that our network model is able to store a large amount of patterns if sufficient neurons are added to the network. (B) Same as in (A), except that the patterns and cues used are half the size as in (A) (i.e. 7 neurons/pattern, 2 neurons/cue). One can observe that memory capacity greatly increases using sparser patterns, as reported to be the case in previous attractor models. (PDF) [file pone.0023113.s002.pdf]

SUPPORTING FIGURE 2

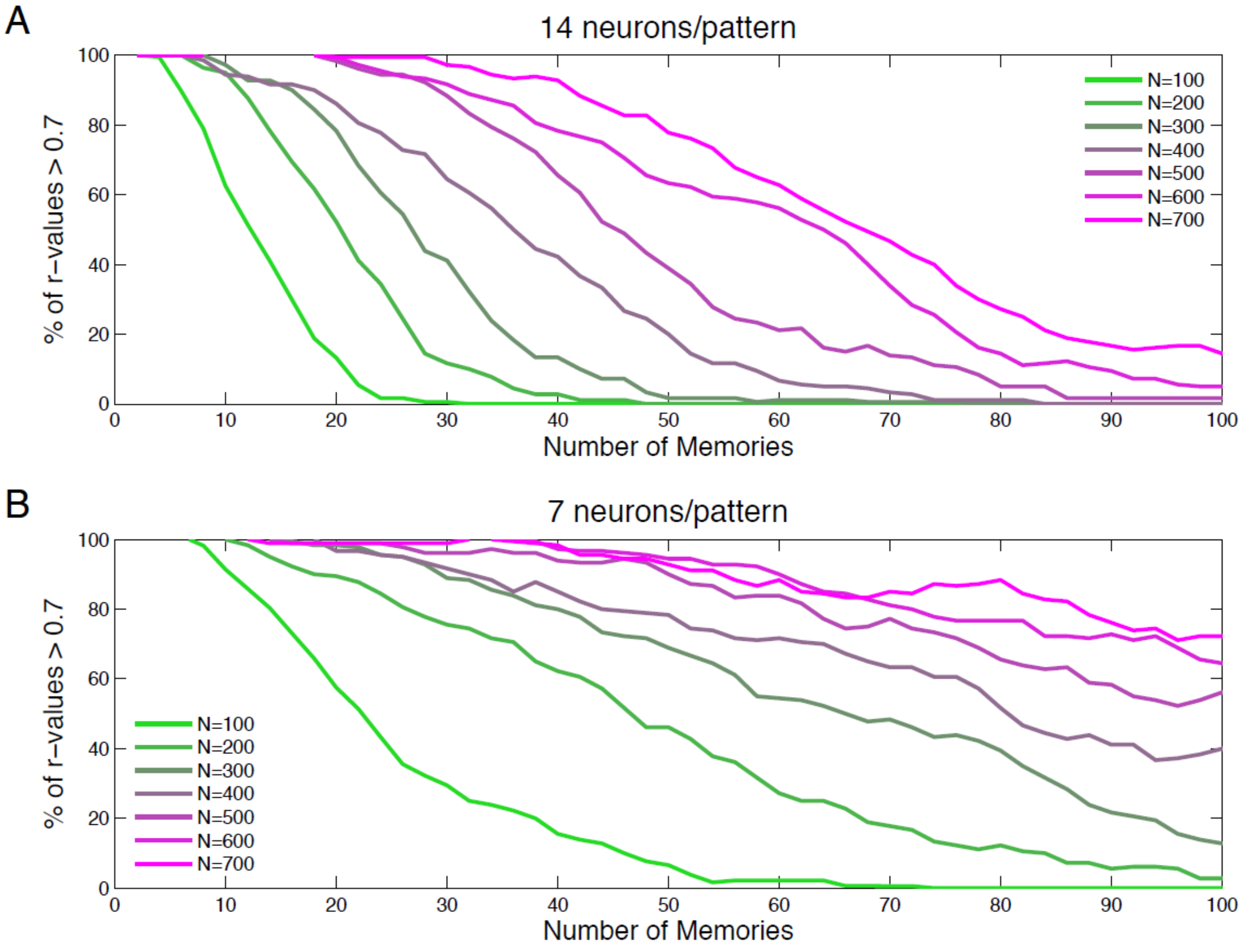

Supporting Figure 2. **Effect of network size and pattern sparseness on memory storage capacity. (A)** Storage capacity of networks with different number of neurons. Different numbers of arbitrary patterns (*x* axis) with random overlap are stored in the synaptic weight matrices W of networks of different sizes, varying from 100 to 700 neurons (color lines), using the same learning rule as in our model (Eq. (3)). Parameters are similar to those used in other simulations, including the size of the memory patterns (14 neurons) and corresponding cues (4 neurons). Retrieval is evaluated by providing the network with a cue pertaining to one of the learned memory patterns and testing the correlation between the activity of neurons in the retrieved pattern and in the original memory pattern, as done by

other authors [92,93]. For each point, 200 retrieval trials using randomly chosen cues and random initial conditions were performed, and the percentage of trials in which successful retrieval occurred (defined as an r value > 0.7 for the correlation) is shown. Memory capacity increases steadily with network size, showing that our network model is able to store a large amount of patterns if sufficient neurons are added to the network **(B)** Same as in (A), except that the patterns and cues used are half the size as in (A) (i.e. 7 neurons/pattern, 2 neurons/cue). One can observe that memory capacity greatly increases using sparser patterns, as reported to be the case in previous attractor models.
