# Supplementary material for: A Mismatch-Based Model for Memory Reconsolidation and Extinction in Attractor Networks
Source: PLoS One. 2011 Aug 3;6(8):e23113. doi: 10.1371/journal.pone.0023113 (PMC3149635; doi:10.1371/journal.pone.0023113)
Supplement: Figure S3 — Comparison of relative memory strengths after single session and multiple session extinction. (A) Basins of attraction after single session extinction. Learning of memories 1, 2 and 3 occurs as in Figure 2C, with extinction learned in a single reexposure session with t = 10. Energy landscape shows relative basins of attraction after learning of the 3 memories. The energy minimum for memory 2 persists, but is higher than that of memory 3, leading to behavioral dominance of the extinction memory. (B) Basins of attraction after multiple session extinction. Learning of memories 1 and 2 occurs as in (A), while extinction occurs through 6 reexposure sessions with t = 6 as in Figure 2D. The energy minimum for memory 2 is significantly reduced by multiple sessions of extinction when compared to a single session, due to the effects of mismatch-induced degradation. This could be related to the finding that spaced extinction trials can lead to less spontaneous recovery and renewal of the original memory than massed trials [94]. (PDF) [file pone.0023113.s003.pdf]

SUPPORTING FIGURE 3

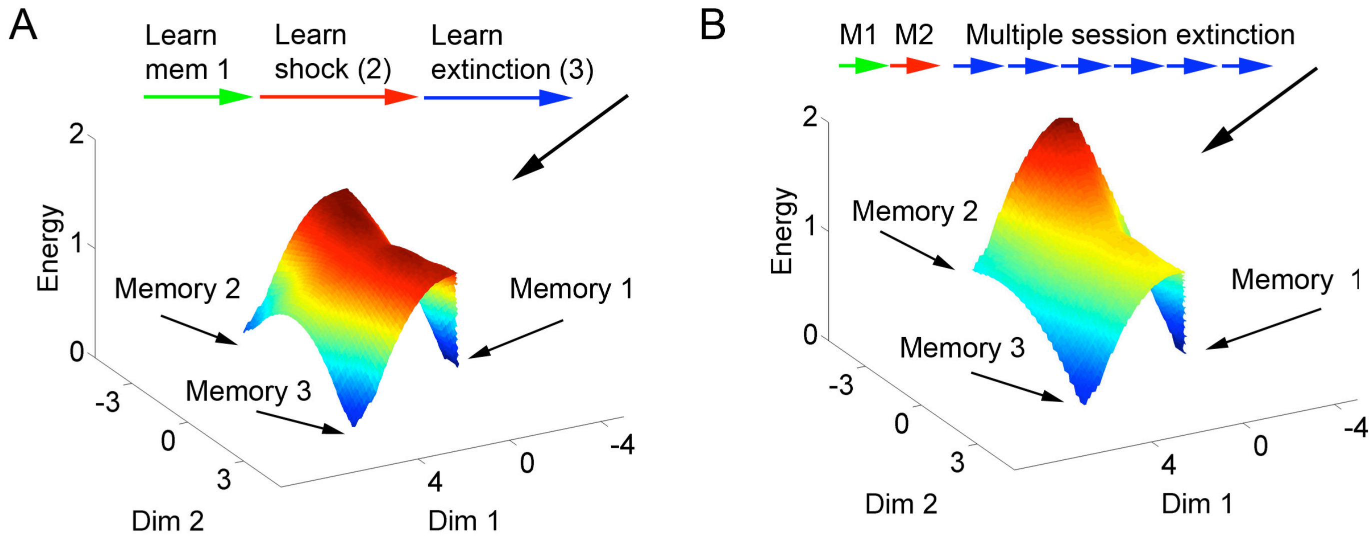


**Supporting Figure 3. Comparison of relative memory strengths after single session and multiple session extinction. (A)** Basins of attraction after single session extinction. Learning of memories 1, 2 and 3 occurs as in Figure 2C, with extinction learned in a single reexposure session with $t$ = 10. Energy landscape shows relative basins of attraction after learning of the 3 memories. The energy minimum for memory 2 persists, but is higher than that of memory 3, leading to behavioral dominance of the extinction memory. **(B)** Basins of attraction after multiple session extinction. Learning of memories 1 and 2 occurs as in (A), while extinction occurs through 6 reexposure sessions with $t$ = 6 as in Figure 2D. The energy minimum for memory 2 is significantly reduced by multiple sessions of extinction when compared to a single session, due to the effects of mismatch-induced degradation. This could be related to the finding that spaced extinction trials can lead to less spontaneous recovery and renewal of the original memory than massed trials [94].
