# Supplementary material for: A Mismatch-Based Model for Memory Reconsolidation and Extinction in Attractor Networks
Source: PLoS One. 2011 Aug 3;6(8):e23113. doi: 10.1371/journal.pone.0023113 (PMC3149635; doi:10.1371/journal.pone.0023113)
Supplement: Figure S4 — Effect of pattern overlap on the development of reconsolidation and extinction. (A) Freezing rates in a retrieval test performed after a reexposure session of variable duration in control conditions (S = 0.8) with different degrees of overlap between memory 2 and memory 3 used in the simulations. The percentage of coactive neurons in both patterns is indicated in the y axis, while the duration of reexposure (t) is indicated in the x axis, with the color scale representing freezing at a subsequent retrieval test. One can observe that above a certain degree of overlap (around 30% of active neurons in common), extinction does not occur with the parameters used in the simulations, suggesting that some degree of pattern separation is necessary for extinction learning. (B) Freezing rates after reexposure of various durations under anisomycin (S = 0). Blockade of HLP shows that reconsolidation blockade occurs in a wider range of overlap (from 7 to 80% of active neurons, approximately) than extinction blockade, which is only observed with up to 30% overlap, as in (A). (PDF) [file pone.0023113.s004.pdf]

SUPPORTING FIGURE 4

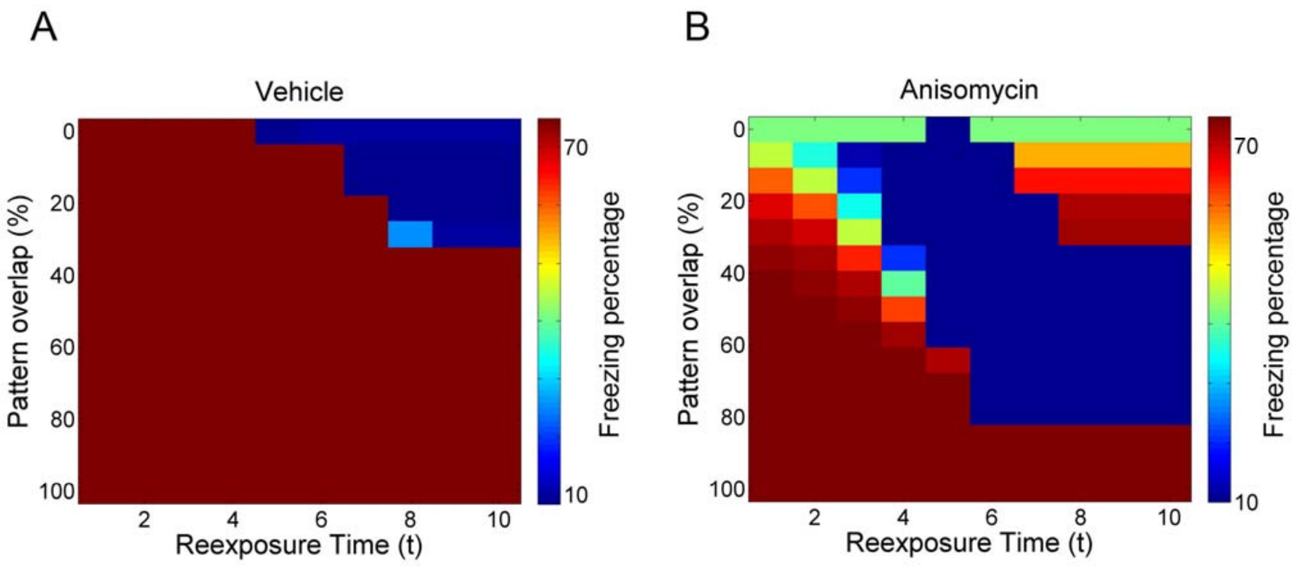

Supporting Figure 4. Effect of pattern overlap on the development of reconsolidation and extinction. **(A)** Freezing rates in a retrieval test performed after a reexposure session of variable duration in control conditions (*S* = 0.8) with different degrees of overlap between memory 2 and memory 3 used in the simulations. The percentage of coactive neurons in both patterns is indicated in the *y* axis, while the duration of reexposure (*t*) is indicated in the *x* axis, with the color scale representing freezing at a subsequent retrieval test. One can observe that above a certain degree of overlap (around 30% of active neurons in common), extinction does not occur with the parameters used in the simulations, suggesting that some degree of pattern separation is necessary for extinction learning. **(B)** Freezing rates after reexposure of various durations under anisomycin (*S* = 0). Blockade of *HLP* shows that reconsolidation blockade occurs in a wider range of overlap (from 7 to 80% of active neurons, approximately) than extinction blockade, which is only observed with up to 30% overlap, as in (A).
