# Supplementary material for: A Mismatch-Based Model for Memory Reconsolidation and Extinction in Attractor Networks
Source: PLoS One. 2011 Aug 3;6(8):e23113. doi: 10.1371/journal.pone.0023113 (PMC3149635; doi:10.1371/journal.pone.0023113)
Supplement: Figure S5 — CA3-CA1 model for mismatch detection, reconsolidation and extinction. (A) Model scheme. Output from the entorhinal cortex (EC) reaches both the CA3 and CA1 regions, providing information on the current context. CA3 neurons possess autoassociative connections, and send information on their retrieved attractor to CA1. (B) Initial learning, reconsolidation and extinction of aversive memories in the CA3-CA1 model. Left column shows a more detailed view of the model scheme, with sample neurons representing the context (square), shock (circle) and absence of shock (triangle). Middle column shows activation of the same neurons during initial learning, reconsolidation and extinction, while the right column shows the synaptic weight changes caused by this activation. (I) Initial learning. Context and shock neurons are activated in all three networks (middle column), leading to strengthening of synapses (solid arrows) between coactive neurons (both in CA3 collaterals and in CA3-CA1 synapses) and inhibition of non-shock neurons in CA3 (circle-capped lines), as shown in the right column. (II) Reconsolidation. Ambiguous information from the EC leads to partial activation of shock and non-shock neurons in CA1, while CA3 still retrieves the original pattern (middle column). The mismatch generated between CA3 and CA1 shock neurons leads to mismatch-induced degradation of their connections (discontinuous arrow), which is compensated by Hebbian learning both within CA3 and in CA3-CA1 connections. (III) Extinction. Cue patterns indicating absence of shock lead to instatement of this pattern both in CA1 and CA3 (middle column). Synaptic weight changes show formation of a new attractor representing extinction in CA3 and strengthening of connections between non-shock neurons in CA3 and CA1, while connections between shock neurons remain unaltered. (C) Effect of anisomycin in reexposure sessions of various durations in the CA3-CA1 model. The x axis represents reexposure duration, while th [file pone.0023113.s005.pdf]

SUPPORTING FIGURE 5

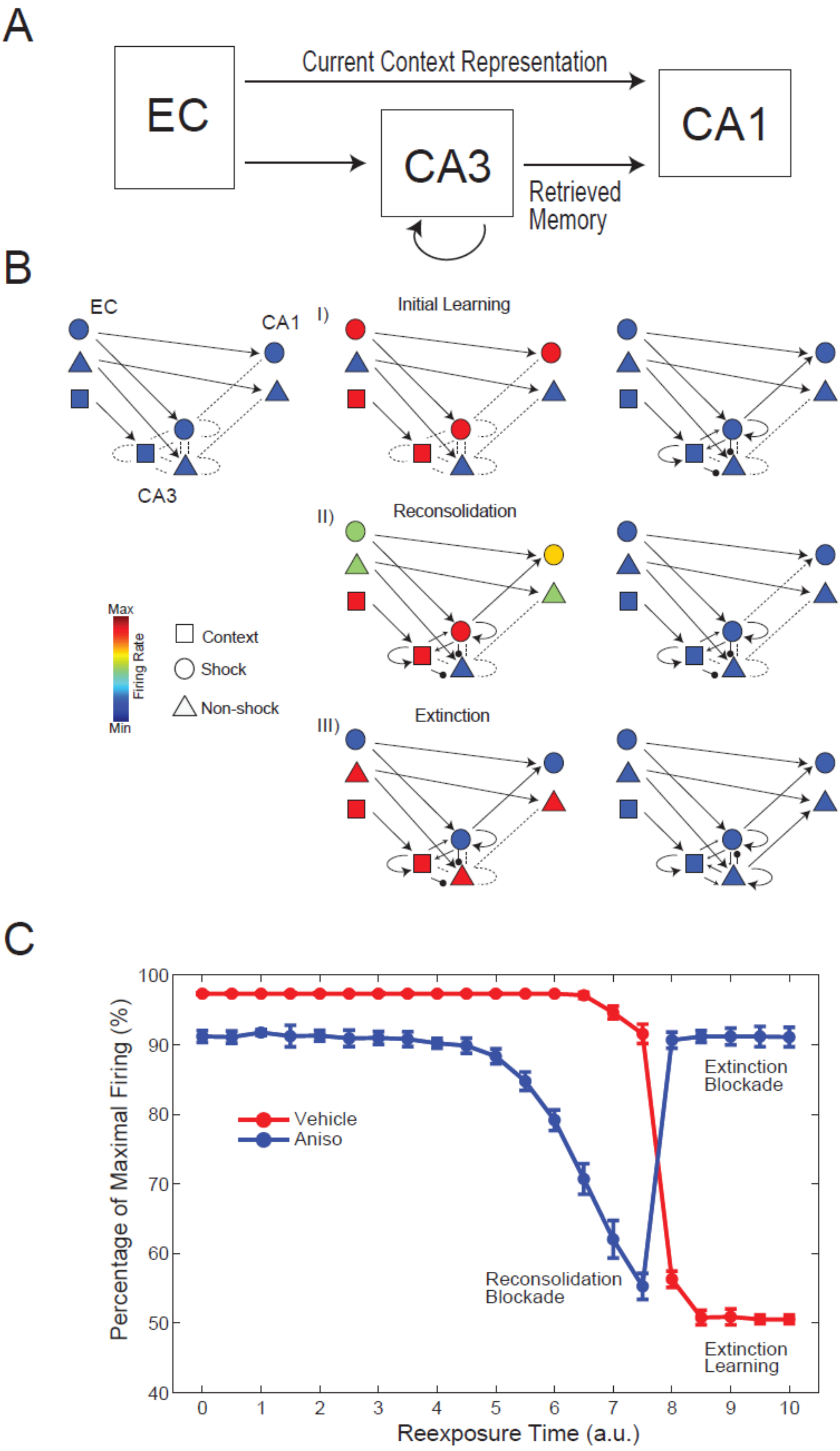

Supporting Figure 5. CA3-CA1 model for mismatch detection, reconsolidation and extinction.

(A) Model scheme. Output from the entorhinal cortex (EC) reaches both the CA3 and CA1 regions, providing information on the current context. CA3 neurons possess autoassociative connections, and send information on their retrieved attractor to CA1. **(B)** Initial learning, reconsolidation and extinction of aversive memories in the CA3-CA1 model. Left column shows a more detailed view of the model scheme, with sample neurons representing the context (square), shock (circle) and absence of shock (triangle). Middle column shows activation of the same neurons during initial learning, reconsolidation and extinction, while the right column shows the synaptic weight changes caused by this activation. (I) Initial learning. Context and shock neurons are activated in all three networks (middle column), leading to strengthening of synapses (solid arrows) between coactive neurons (both in CA3 collaterals and in CA3-CA1 synapses) and inhibition of non-shock neurons in CA3 (circle-capped lines), as shown in the right column. (II) Reconsolidation. Ambiguous information from the EC leads to partial activation of shock and non-shock neurons in CA1, while CA3 still retrieves the original pattern (middle column). The mismatch generated between CA3 and CA1 shock neurons leads to mismatch-induced degradation of their connections (discontinuous arrow), which is compensated by Hebbian learning both within CA3 and in CA3-CA1 connections. (III) Extinction. Cue patterns indicating absence of shock lead to instatement of this pattern both in CA1 and CA3 (middle column). Synaptic weight changes show formation of a new attractor representing extinction in CA3 and strengthening of connections between non-shock neurons in CA3 and CA1, while connections between shock neurons remain unaltered. **(C)** Effect of anisomycin in reexposure sessions of various durations in the CA3-CA1 model. The x axis represents reexposure duration, while the lines show freezing percentages (y axis) of vehicle (red) and anisomycin (blue) groups in retrieval

tests performed after reexposure. The dependence of retrieval on reexposure duration in both groups is qualitatively similar to what is observed with the general model in Figure 3F.
