# Supplementary material for: A Mismatch-Based Model for Memory Reconsolidation and Extinction in Attractor Networks
Source: PLoS One. 2011 Aug 3;6(8):e23113. doi: 10.1371/journal.pone.0023113 (PMC3149635; doi:10.1371/journal.pone.0023113)
Supplement: Figure S6 — Selectivity of reconsolidation blockade to the retrieved attractor. (A) Reconsolidation of fear conditioning. Bars show freezing percentages after learning memories 1 and 2 (left) and after a subsequent reexposure session with t = 5 (right) in animals treated with vehicle and anisomycin in reexposure. As in Figure 3C, freezing remains high after reexposure in the vehicle group, but decreases due to reconsolidation blockade in the anisomycin group. (B) Reconsolidation of fear extinction. Left bars show freezing after learning of memories 1 and 2 followed by fear extinction with a long reexposure session (t = 10), which leads to learning of memory 3. As occurs in Figures 2C and 3C, memory 3 is dominant after this protocol, leading to low freezing. Subsequently, a reexposure session with the same duration as in (A) is performed; vehicle or anisomycin is administered in this session, and right bars show freezing in a subsequent retrieval test. Freezing remains low in the vehicle group, but increases in the anisomycin treated group, indicating that the extinction memory, which was preferentially retrieved during reexposure, becomes susceptible to reconsolidation blockade. (C) Energy landscapes showing relative basins of attraction for memories 1, 2 and 3 after learning of memories 1 and 2 (left) and after a subsequent reexposure session (t = 5) with vehicle (middle) or anisomycin (right). Right panel shows a decrease in the basin of attraction of memory 2 (shock memory). (D) Energy landscapes showing relative basins of attraction for memories 1, 2 and 3 after learning of memories 1, 2 and 3 (left) and after a subsequent reexposure session (t = 5) with vehicle (middle) or anisomycin (right). Right panel shows a decrease in the basin of attraction of memory 3 (extinction memory). (PDF) [file pone.0023113.s006.pdf]

SUPPORTING FIGURE 6

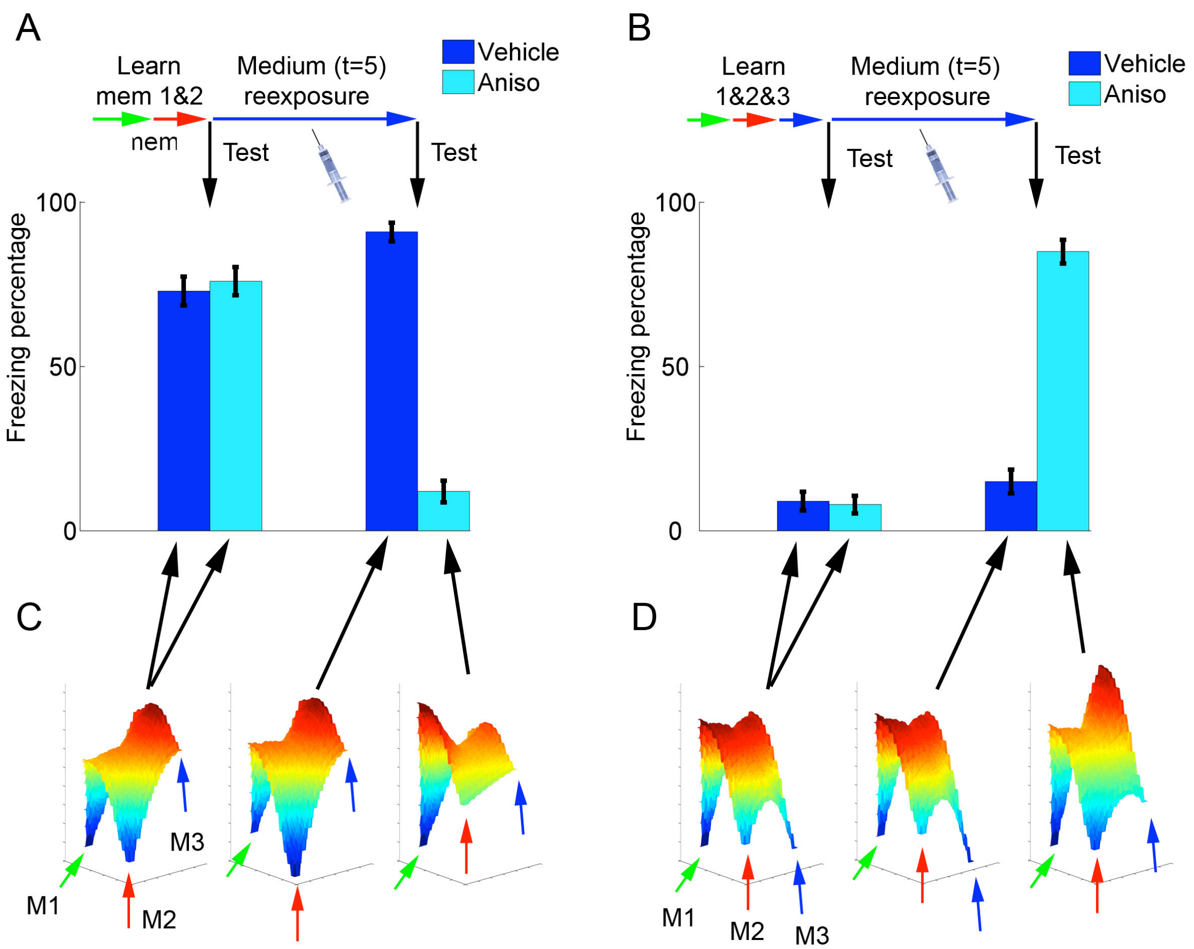

Supporting Figure 6. Selectivity of reconsolidation blockade to the retrieved attractor. (A) Reconsolidation of fear conditioning. Bars show freezing percentages after learning memories 1 and 2 (left) and after a subsequent reexposure session with t = 5 (right) in animals treated with vehicle and anisomycin in reexposure. As in Figure 3C, freezing remains high after reexposure in the vehicle group, but decreases due to reconsolidation blockade in the anisomycin group. **(B)** Reconsolidation of fear extinction. Left bars show freezing after learning of memories 1 and 2 followed by fear extinction with a long reexposure session (t = 10), which leads to learning of memory 3. As occurs in Figures 2C and 3C, memory 3 is dominant after this protocol, leading to low freezing. Subsequently, a reexposure session with the same duration as in (A) is performed; vehicle or anisomycin is

administered in this session, and right bars show freezing in a subsequent retrieval test. Freezing remains low in the vehicle group, but increases in the anisomycin treated group, indicating that the extinction memory, which was preferentially retrieved during reexposure, becomes susceptible to reconsolidation blockade. **(C)** Energy landscapes showing relative basins of attraction for memories 1, 2 and 3 after learning of memories 1 and 2 (left) and after a subsequent reexposure session (t = 5) with vehicle (middle) or anisomycin (right). Right panel shows a decrease in the basin of attraction of memory 2 (shock memory). **(D)** Energy landscapes showing relative basins of attraction for memories 1, 2 and 3 after learning of memories 1, 2 and 3 (left) and after a subsequent reexposure session (t = 5) with vehicle (middle) or anisomycin (right). Right panel shows a decrease in the basin of attraction of memory 3 (extinction memory).
