# Supplementary material for: A Mismatch-Based Model for Memory Reconsolidation and Extinction in Attractor Networks
Source: PLoS One. 2011 Aug 3;6(8):e23113. doi: 10.1371/journal.pone.0023113 (PMC3149635; doi:10.1371/journal.pone.0023113)
Supplement: Figure S7 — Differential effects of enhanced mismatch-induced degradation on reconsolidation and multiple session extinction. (A) After learning of memory 2, increasing D from 1.25 (blue bar) to 1.5 (orange bar) during a reexposure session (t = 7.5) leads to a decrease in freezing as compared to the vehicle group, as the reinforcement of memory 2 is impaired. (B) In a multiple session extinction protocol (6 sessions with t = 6), the same increase in D used in (A) leads to an acceleration of memory extinction. (PDF) [file pone.0023113.s007.pdf]

SUPPORTING FIGURE 7

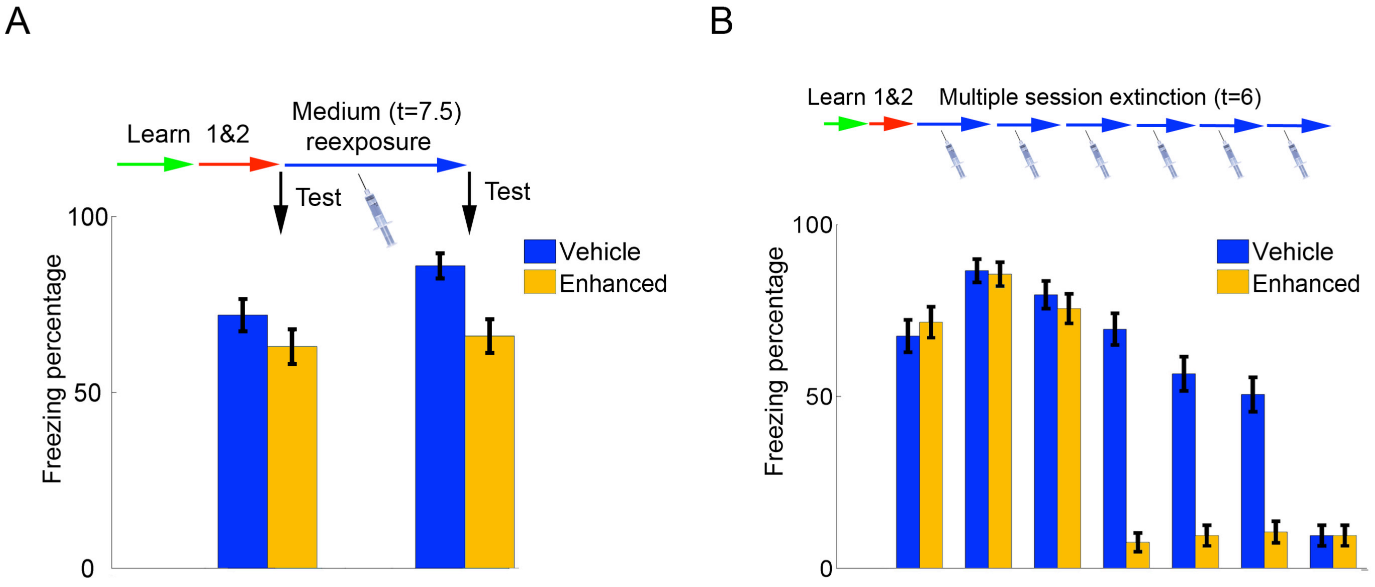

Supporting Figure 7. Differential effects of enhanced mismatch-induced degradation on reconsolidation and multiple session extinction. (A) After learning of memory 2, increasing D from 1.25 (blue bar) to 1.5 (orange bar) during a reexposure session (t = 7.5) leads to a decrease in freezing as compared to the vehicle group, as the normal reinforcement of memory 2 is blocked. **(B)** In a multiple session extinction protocol (6 sessions with t = 6), the same increase in D used in (A) leads to an acceleration of memory extinction.
