# Supplementary material for: A Mismatch-Based Model for Memory Reconsolidation and Extinction in Attractor Networks
Source: PLoS One. 2011 Aug 3;6(8):e23113. doi: 10.1371/journal.pone.0023113 (PMC3149635; doi:10.1371/journal.pone.0023113)
Supplement: Figure S8 — Effect of S and D variations on the development of reconsolidation and extinction. (A) Freezing rates in a retrieval test performed after a reexposure session of variable duration (x axis) with different values of S (y axis) used throughout the simulations (i.e. learning of memories 1 and 2 and reexposure sessions), while D is fixed at 1.25. One can observe that, with the other parameters kept fixed, extinction happens for S values varying from 0.6 to 0.9, although at different reexposure durations. (B) Freezing rates retrieved as in (A), but with anisomycin administration (S = 0) simulated during reexposure, with different values of S used during learning of memories 1 and 2. Reconsolidation occurs for values of S varying from 0.7 to 1, albeit at variable reexposure durations. (C) Freezing rates retrieved as in (A) and (B), but with different values of D throughout the simulations, while S is fixed at 0.8. D values have little effect on the occurrence of extinction, as there is no mismatch between current context and retrieved attractors in extinction conditions; however, high values of D (i.e. above 1.6) can lead to an amnestic effect under reconsolidation conditions, even in the absence of anisomycin. (D) Freezing rates retrieved as in (C), but with anisomycin administration during reexposure. Reconsolidation blockade is observed at all values of D included in the simulations, although higher values lead to amnesia at progressively shorter reexposure durations. (PDF) [file pone.0023113.s008.pdf]

SUPPORTING FIGURE 8

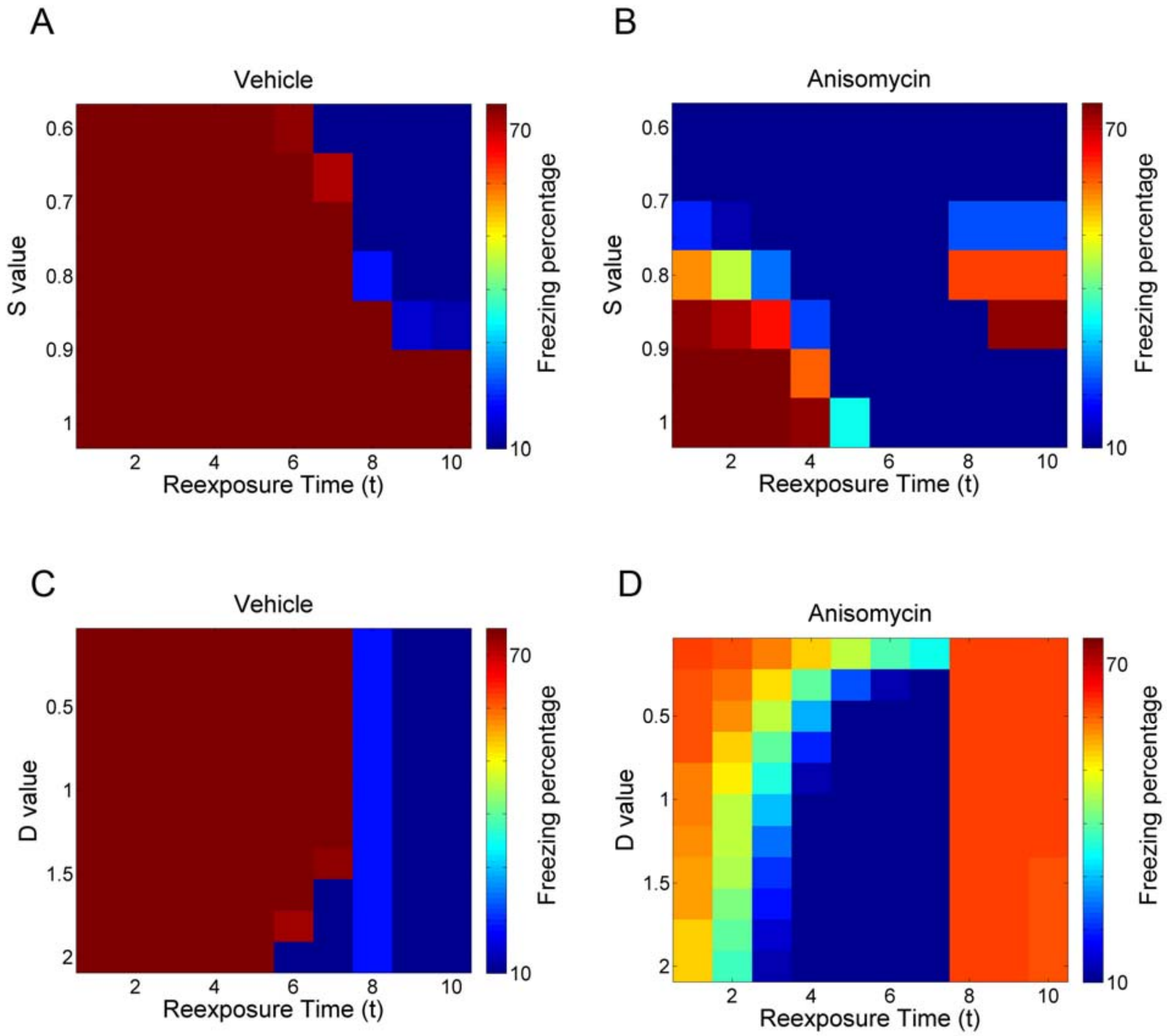

Supporting Figure 8. Effect of *S* and *D* variations on the development of reconsolidation and extinction. (A) Freezing rates in a retrieval test performed after a reexposure session of variable duration (*x* axis) with different values of *S* (*y* axis) used throughout the simulations (i.e. learning of memories 1 and 2 and reexposure sessions), while *D* is fixed at 1.25. One can observe that, with the other parameters kept fixed, extinction happens for *S* values varying from 0.6 to 0.9, although at different reexposure durations. **(B)** Freezing rates retrieved as in (A), but with anisomycin administration (*S* = 0) simulated during reexposure, with different values of *S* used during learning of memories 1

and 2. Reconsolidation occurs for values of *S* varying from 0.7 to 1, albeit at variable reexposure durations. **(C)** Freezing rates retrieved as in (A) and (B), but with different values of *D* throughout the simulations, while *S* is fixed at 0.8. *D* values have little effect on the occurrence of extinction, as there is no mismatch between current context and retrieved attractors in extinction conditions; however, high values of *D* (i.e. above 1.6) can lead to an amnestic effect under reconsolidation conditions, even in the absence of anisomycin. **(D)** Freezing rates retrieved as in (C), but with anisomycin administration during reexposure. Reconsolidation blockade is observed at all values of *D* included in the simulations, although higher values lead to amnesia at progressively shorter reexposure durations.
