# Supplementary material for: A Mismatch-Based Model for Memory Reconsolidation and Extinction in Attractor Networks
Source: PLoS One. 2011 Aug 3;6(8):e23113. doi: 10.1371/journal.pone.0023113 (PMC3149635; doi:10.1371/journal.pone.0023113)
Supplement: Text S1 — (PDF) [file pone.0023113.s009.pdf]

SUPPORTING TEXT

CA3-CA1 model for mismatch detection, reconsolidation and extinction

To illustrate how mismatch detection (which underlies the phenomenon of reconsolidation in our model) could be achieved using the general connectivity structure of the hippocampus, we present a model in which the dual inputs received by CA1 neurons, which convey information on both retrieved memories (from the CA3 region) and current representations of the context (from the entorhinal cortex), allow this function to be performed. Such a model is inspired by previous work which has proposed such a mismatch-detecting circuit in more detail (Hasselmo and Wyble, 1997; Lisman, 1999; Lisman and Grace, 2005), although to our knowledge this mismatch detection has not been related to reconsolidation or extinction in former computational models.

Model description

The networks representing CA3 and CA1 are composed of 100 neurons each. A cue network, meant to represent input from the entorhinal cortex (EC) sends one-to-one connections to both the CA3 and CA1 regions; the CA3 region sends one-to-one connections to CA1, and also presents all-to-all recurrent connections (Figure S5A). For the sake of simplicity in correlating activity in CA1 with retrieval of attractors in CA3, we do not model the dentate gyrus as a relay between the EC and CA3, as this would imply in the orthogonalization of patterns and make ensemble representations distinct in CA3 and CA1. Nevertheless, such distinctions in the representation of information in CA3 and CA1 certainly exist in the rodent hippocampus, as shown by experimental evidence (Leutgeb et al., 2004).

In this formulation, the EC corresponds to the cue network already introduced; it provides inputs carrying information about the current context representation to both CA3 and CA1. Neurons in CA3 and CA1, meanwhile, follow the dynamics described in Eq (1). Hebbian learning (*HLP*) occurs in CA3-CA3 synapses following the same formalism described in Eq (3). Notice, therefore, that the EC-CA3 subnetwork corresponds to the same cue-attractor network employed in the general model, with the exception that mismatch-induced degradation does not occur in the attractor network (CA3) itself, but rather in the synapses between CA3 and CA1 (see below), as recently suggested by evidence from electrophysiological recordings of the hippocampus after reexposure to a learning context (Clarke et al., 2010).

Through Hebbian learning, there is strengthening of the connections in a subset of the recurrent collaterals in CA3; namely, connections within and among CA3 shock and context neurons get reinforced (similarly to what occurs in the general model). In addition, heteroassociative plasticity through Hebbian learning is modelled to occur in CA3-CA1 synapses. Thus, upon learning of the shock memory, there is concomitant activation of neurons representing the shock in both CA3 and CA1 due to inputs from the EC (Figure S5B, top middle panel), leading to a strengthening of the connections from CA3 shock neurons to CA1 shock neurons (Figure S5B, top right panel).

During later retrieval tests, when only the context is presented, the CA3 attractor network will complete the pattern by activating shock neurons, and these will activate the corresponding neurons in CA1 through the connections which were reinforced during the initial learning. Retrieval of the fear memory in this model is tested by measuring the activity of shock neurons in CA1 (which represents the output of the hippocampus) in response to the context cue, and is dependent on Hebbian

learning both in CA3 recurrent synapses and in CA3-CA1 connections, as suggested by most models of hippocampal physiology (Rolls and Kesner, 2006; Rolls, 2007).

Since the CA1 region receives inputs from both the EC and the CA3 region, it has been proposed to play a role in detecting mismatch between stored information (arriving from CA3) and current sensory/contextual representations (arriving from the EC) (Hasselmo and Wyble, 1997; Lisman and Grace, 2005). Based on this, in this formulation we model mismatch-induced degradation as occurring in synapses from CA3 to CA1, in accordance with recent evidence showing transient decay of EPSPs in these connections after reexposure to a learning context (Clarke et al., 2010). *MID* is triggered in these synapses, thus, whenever activity is higher presynaptically than postsynaptically (that is, when CA3 neurons fire at a higher frequency than the CA1 neurons they connect to, indicating mismatch between information from CA3 and the EC). This degradation term acts to reduce the synaptic strength from CA3 to CA1 in response to mismatch**.**

Therefore, in contextual reexposure of intermediate durations (i.e. reconsolidation conditions), when the EC sends ambiguous information partially activating both shock and non-shock neurons in CA1, the activity of shock neurons in CA1 will be in disagreement with those in CA3, which were fully activated thanks to attractor functioning in the recurrent collaterals (Figure S5B, middle panel). This will lead to weakening of the synaptic weights between shock neurons in CA3 and CA1 due to *MID* (represented by the dashed arrow in Figure S5B, middle right panel). In control conditions, such an effect is counterbalanced (partially or totally) because the same synapses also present Hebbian learning (since both CA1 and CA3 shock neurons are active). The degradation effect prevails, however, when the effect of anisomycin is simulated. In this case, as there is no Hebbian learning (which depends

on protein synthesis), synaptic strength from CA3 to CA1 shock neurons decreases through *MID* and reconsolidation blockade is observed. On the other hand, for longer reexposure times, there is no mismatch between CA3 and CA1, as the cue pattern is distinct enough to form a new attractor in CA3, which matches the current representation in the EC. In this case, the CA3 and CA1 regions exhibit similar patterns during reexposure, and extinction learning occurs. As in the simple cue-attractor network, extinction learning can be prevented by blocking protein synthesis, in which case the original memory is preserved.

Figure S5C shows the results obtained with this kind of network, which are qualitatively similar to those obtained with our general, non-topological model (see Figure 3F). Thus, simple retrieval, reconsolidation and extinction (as well as the different effects of anisomycin in these conditions), can be observed in this alternative implementation. This shows that the mechanism of mismatch detection could be either implemented within the autoassociative connections of an attractor-like network (such as CA3) or in a separate set of heteroassociative connections (such as CA3-CA1 synapses). To distinguish between these possibilities, more specific pharmacological studies targeting distinct regions of the hippocampus in both reconsolidation and extinction are necessary. An interesting fact, however, is that inhibitors of protein degradation through the ubiquitin-proteasome system prevent reconsolidation blockade when injected in CA1 (Lee et al., 2008), but can actually promote it when injected in CA3 (Artinian et al., 2008). This suggests that, at least in the rodent hippocampus, the protein degradation-related processes underlying memory destabilization might be indeed happening in the CA1 region, as proposed in the present model.

Methods

The details of the model implementation are as follows: the EC-CA3 subnetwork is modelled exactly as our general model (where EC corresponds to the cue network, and CA3 is the attractor network), and the same default parameters were used with the exception that D is set to 0 within the attractor network (in particular, $\tau = 1$, $N = 100$, $\gamma = 0.15$, $s_0 = 1$ and $S = 0.8$). In addition, we model a CA1 network, which is also composed of $N = 100$ neurons and receives one-to-one connections from both CA3 and EC. That is, neurons in CA1 follow Eq (1) and their external current is given by $I = w_{CA3}u_{CA3} + w_{EC}u_{EC}$, where u_{EC} and u_{CA3} refer to the activity of neurons in CA3 and EC, respectively. Note that u_{CA3} refers to the steady state activity achieved by neurons in CA3 upon presentation of the cue currents (EC), whereas u_{EC} refers to the same cue currents which reach CA3 (that is, u_{EC} can assume the form of I_1, I_2, I_3, or a mix pattern of I_2 and I_3 (see Eq (5)); the factors w_{CA3} and w_{EC} determine the weight of these connections. We assume that w_{CA3} is subject to both Hebbian synaptic strengthening and mismatch-induced synaptic weakening (see below), whereas w_{EC} is constant. After the steady state in CA1 is achieved, w_{CA3} is updated according to the following rule:

$$\Delta w_{CA3} = -\gamma w_{CA3} + HLP_{CA3CA1} + MID_{CA3CA1} \quad \text{(S1)}$$

where HLP_{CA3CA1} represents the Hebbian learning factor between CA3 and CA1 cells and is given by

$$HLP_{CA3CA1} = S_{CA1CA3}\, u_{CA1}\, u_{CA3} \quad \text{(S2)}$$

while MID is the mismatch-induced degradation of synaptic weights between CA3 and CA1 neurons and is defined by:

$$MID_{CA3CA1} = -D_{CA1CA3}\, max(u_{CA3} - u_{CA1}, 0) \quad \text{(S3)}$$

For these simulations, we used γ=0.15, S_{CA1CA3} = 1.8, D_{CA1CA3} = 3, w_{EC} = 1.25. Synaptic weights from CA3 to CA1 start at 0 (i.e., w_{CA3} = 0) and evolve according the equations above, with a saturation value of 1.8.

Learning of memory patterns 1, 2 and 3 (or mixed patterns) occurs exactly as in the general model, that is, by having the EC providing the corresponding cue inputs. Retrieval of the shock memory is tested by computing the mean activity of CA1 shock neurons in response to a cue consisting solely of the context neurons in the EC, while other cue inputs are set to 0.

References

Artinian J, McGauran AT, De Jaeger X, Mouledous L, Frances B, et al. (2008) Protein degradation, as with protein synthesis, is required during not only long-term spatial memory consolidation but also reconsolidation. Eur J Neurosci 27: 3009-3019.

Clarke JR, Cammarota M, Gruart A, Izquierdo I, Delgado-Garcia J-M (2010) Plastic modifications induced by object recognition memory processing. Proceedings of the National Academy of Sciences 107: 2652-2657.

Hasselmo ME, Wyble BP (1997) Free recall and recognition in a network model of the hippocampus: simulating effects of scopolamine on human memory function. Behav Brain Res 89: 1-34.

Lee SH, Choi JH, Lee N, Lee HR, Kim JI, et al. (2008) Synaptic protein degradation underlies destabilization of retrieved fear memory. Science 319: 1253-1256.

Leutgeb S, Leutgeb JK, Treves A, Moser MB, Moser EI (2004) Distinct ensemble codes in hippocampal areas CA3 and CA1. Science 305: 1295-1298.

Lisman JE (1999) Relating hippocampal circuitry to function: recall of memory sequences by reciprocal dentate-CA3 interactions. Neuron 22: 233-242.

Lisman JE, Grace AA (2005) The hippocampal-VTA loop: controlling the entry of information into long-term memory. Neuron 46: 703-713.

Rolls ET (2007) An attractor network in the hippocampus: theory and neurophysiology. Learn Mem 14: 714-731.

Rolls ET, Kesner RP (2006) A computational theory of hippocampal function, and empirical tests of the theory. Prog Neurobiol 79: 1-48.
